# Vulnerability of Labile Organic Matter to Eutrophication and Warming in Temperate Mangrove Ecosystems

**DOI:** 10.1111/gcb.70087

**Published:** 2025-02-14

**Authors:** Timothy Thomson, Conrad A. Pilditch, Marco Fusi, Natalie Prinz, Carolyn J. Lundquist, Joanne I. Ellis

**Affiliations:** ^1^ School of Science University of Waikato Tauranga New Zealand; ^2^ Insitute of Marine Sciences University of Auckland, Science Centre Auckland New Zealand; ^3^ School of Environment University of Auckland, Science Centre Auckland New Zealand; ^4^ Dove Marine Laboratory School of Natural and Environmental Sciences Newcastle University Newcastle UK; ^5^ National Institute of Water & Atmospheric Research Hamilton New Zealand

**Keywords:** anthropogenic stressors, carbon cycling, carbon‐climate feedback, coastal biogeochemistry, decomposition, tea bag index

## Abstract

The sediments in mangrove forests play an important role in the global carbon cycle due to high inputs of organic matter (OM) and low decomposition rates, making them highly efficient at sequestering carbon. The balance between OM sequestration and decomposition in these systems is influenced by a complex interplay of environmental factors. However, there is a large amount of uncertainty surrounding decomposition rates from mangrove forests, particularly at regional scales. We used standardized decomposition assays of a labile and recalcitrant substrate in 30 estuaries, spanning a gradient in human land use intensity, to identify dominant drivers of OM decomposition in temperate mangrove forests. Our results reveal that, while labile OM decomposition is strongly driven by eutrophication, recalcitrant OM decomposition is primarily influenced by increases in the minimum sediment temperature. Furthermore, we demonstrate that nutrient enrichment from human land use, in combination with increased sediment temperature, synergistically accelerates the decomposition of labile OM, thereby threatening the carbon sequestration potential of these ecosystems. This suggests that coastal eutrophication can exacerbate the effects of warming on decomposition, leading to heightened vulnerability of carbon storage and potential feedbacks between local and global stressors.

## Graphical abstract

We studied 30 estuaries with varying human impact to understand what drives organic matter (OM) decomposition in temperate mangrove forests. We found that human‐derived nutrient pollution speeds up the breakdown of easily decomposable OM, while increased sediment temperatures accelerate the breakdown of tougher OM. When combined, nutrient pollution and warming may work together to synergistically increase decomposition rates, threatening the ability of mangroves to store carbon. This highlights how local human activities and global climate change can jointly weaken these critical ecosystems, potentially reducing their role in mitigating climate change.

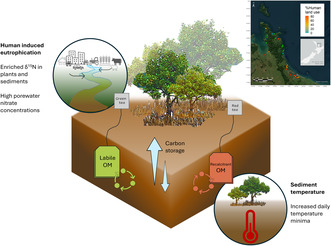

## 1 Introduction

Mangrove forests are among the most efficient ecosystems at sequestering carbon (Alongi 2020a; Donato et al. 2011), due to a combination of high productivity, high sediment retention, and low decomposition rates (Alongi 2020b), leading them to be considered as key habitats for climate change mitigation (Lovelock and Duarte 2019). The capacity of a system to sequester carbon relies on the balance between carbon inputs and losses, and is therefore intrinsically linked to the rate at which organic matter (OM) decomposes (Davidson and Janssens 2006). Factors accelerating the rate of OM decomposition consequently counteract the climate change mitigation potential by driving carbon losses from sediments. OM decomposition in mangrove forests is slow because anoxic conditions are found just below the sediment surface, meaning that sulfate‐reduction and fermentation are the main decomposition pathways (Alongi 1998; Kristensen et al. 2008). However, understanding the environmental factors influencing decomposition in mangrove sediments is an active field of research, and to date empirical data are variable, indicating a high complexity of potential feedbacks and interactions of global and local factors (Friedlingstein et al. 2006; Schmidt et al. 2011; Viscarra Rossel et al. 2019).

A multitude of environmental factors, including climate and organic matter quality, influences OM decomposition. Temperature has been determined as a major driver of OM decomposition on a global scale (Allison et al. 2010; Davidson and Janssens 2006; Nissan et al. 2023), and it is therefore unsurprising that high seasonal variability in decomposition rates has been observed, particularly in temperate environments (Arndt et al. 2013). Leaf litter quality and bio‐available nitrogen are also important factors, with low C:N ratios and high initial tissue nitrogen concentration accelerating decomposition (Djukic et al. 2018; Nordhaus et al. 2017; Parton et al. 2007). Organic matter quality can be variable in mangrove forests, ranging from highly labile compounds associated with microphytobenthic (MPB) production and root exudates to refractory leaf litter and woody fragments (Arndt et al. 2013; Friesen et al. 2018; Kristensen et al. 2008). Local environmental factors, such as shore elevation, affect decomposition of mangrove litter, probably due to variations in leaching rates and microbial dispersal (Middleton and Mckee 2001; Thomson et al. 2022) while complex sediment aggregate structures were found to protect OM from degradation in mangrove sediments (Sun et al. 2019). These studies suggest that the factors controlling OM decomposition in mangrove forests are likely to be temporally and spatially variable. This variability, combined with the dynamic nature of coastal ecosystems, makes it extremely difficult to predict decomposition rates and how they may be impacted by environmental change (Arndt et al. 2013; Feller et al. 2010; Twilley et al. 2019).

Coastal zones are exposed to land‐derived stressors that are exacerbated by anthropogenic activities (Doney 2010; Wakwella et al. 2023). Due to conversion of land for human use, stressors such as nutrient enrichment and sedimentation can impact coastal vegetated habitats (Cloern 2001; Steffen et al. 2015; Wakwella et al. 2023). While the effects of nutrient enrichment on mangrove trees are well studied (Feller et al. 2009), it is unclear how the increased nutrient availability will impact OM decomposition rates in mangrove sediments, which may have implications for carbon losses from the system (Macreadie et al. 2012). Several studies have suggested that nutrient enrichment increases organic matter quality by increasing leaf‐to‐below‐ground biomass production, lowering the C:N ratio of plant tissues, and reducing nutrient retention rates from senescent leaves (Gritcan et al. 2016; Liu et al. 2024; Mckee 1995; Naidoo 2009; Reef et al. 2010). This suggests that eutrophication may contribute to accelerated OM decomposition in mangrove sediments.

However, contrasting evidence supports the inhibitory effects of nutrient enrichment on decomposition of organic material exists (Fog 1988; Janssens et al. 2010; Ouyang et al. 2023; Zang et al. 2024). Nitrogen‐induced carbon limitation (through various pathways, i.e., reduced microbial biomass, reduced root exudates, reduced fine root growth) has been proposed as one mechanism suppressing remineralization of organic material in terrestrial soils (Janssens et al. 2010; Zang et al. 2024). This limitation may be less relevant in mangrove sediments, however, due to a priming effect by microbial primary producers that provide a source of labile carbon to the system (Riekenberg et al. 2018; Zhu et al. 2024). This would reduce the carbon limitation for heterotrophic decomposition and potentially further threaten the loss of stored carbon (Fontaine et al. 2007; Riekenberg et al. 2020). Concordantly, studies in aquatic settings that measured decomposition with standardized assays (i.e., litter bags, cotton strips), rather than soil/sediment respiration, identified nutrient concentration as a driver of increasing organic matter decomposition (Ouyang et al. 2023; Song et al. 2019; Tiegs et al. 2024). To date, the relative effect of global (e.g., temperature increases) and local stressors (e.g., nutrient enrichment) on the ability of mangrove sediments to stabilize OM is poorly understood.

Several studies have assessed the effects of environmental variables, including temperature, nutrient availability, and grain size, on the decomposition rate of organic matter in mangrove forests (Gladstone‐Gallagher et al. 2014; Jessen et al. 2021; Mueller et al. 2018). However, these studies generally focused on single variables that affect decomposition rates, thereby neglecting the complexity of interacting factors that may regulate this process (Schmidt et al. 2011; Stoica et al. 2023). On the other hand, studies that focus on global drivers of change in decomposition rates can be biased by large‐scale patterns that understate the importance of local conditions (Djukic et al. 2018; Mueller et al. 2018; Sarneel et al. 2024). Moreover, very few studies attempt to identify a direct link between anthropogenic stressors, such as eutrophication, and OM decomposition in mangrove forests (Santos‐Andrade et al. 2021; Spivak et al. 2019). Predictions on how the large amounts of carbon stored in mangrove habitats will respond to warming and local environmental stressors are highly uncertain (Spivak et al. 2019). Yet, understanding how fast OM can be either stored or lost is critical not just for understanding climate change impacts on blue carbon ecosystems, but also for sustaining sediment ecosystem function.

To address these knowledge gaps, we applied a field‐based approach, selecting 30 sites along a broad land use intensity gradient. Previously we showed that catchment land use influenced forest structure and sediment biogeochemistry across this gradient (Thomson et al. 2024). Our aim here was to determine how these changes affect OM decomposition rates in mangrove sediments. To do so, we used a standardized tea bag assay (Keuskamp et al. 2013) with organic material of two differing qualities: a liable “green” and more refractory “red” tea. Although reservations exist as to how accurately this method reflects the decomposition of local litter and how it relates to carbon sequestration, it is a useful way to compare rates across a wide range of sites and settings (Joly et al. 2023; Prescott 2010; Sarneel et al. 2024). By experimentally assessing OM decomposition across a strong human land use gradient, we aim to provide novel insights into how interacting environmental variables and stressors influence decomposition rates in mangrove ecosystems. We hypothesize that sediment temperature will be a dominant driver of organic matter decomposition. Furthermore, we expect nutrient concentrations in the system to be correlated with organic matter decomposition and that this impact will be reflected along the gradient of land use intensity.

## 2 Methods

### 2.1 Study Design

**FIGURE 1:**
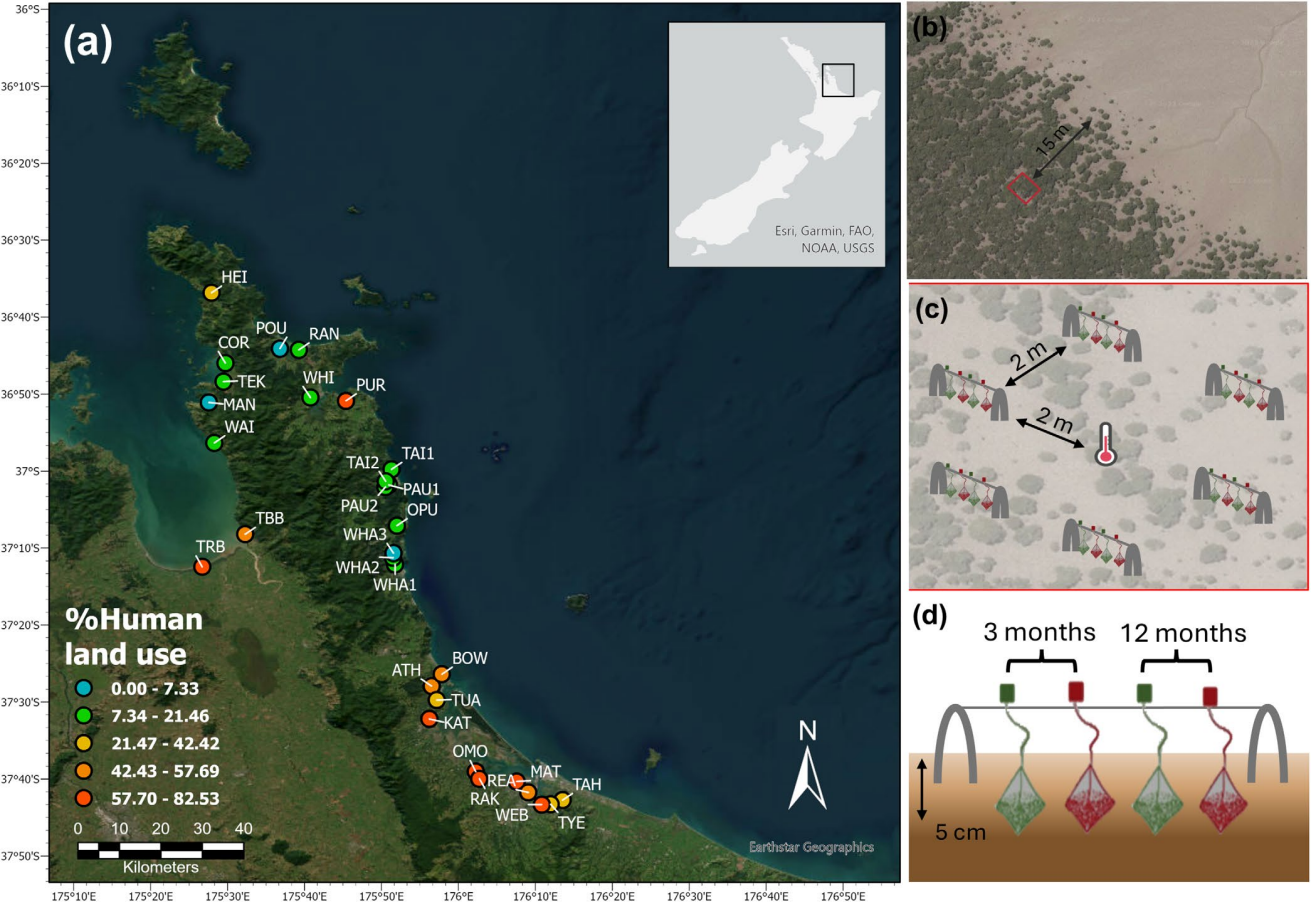
Spatial distribution of sites along a catchment land use gradient and experimental design at each site. (a) The distribution of the sites along the east coast of the North Island of New Zealand along a land use gradient (adapted from Thomson et al. 2024), and (b) the location of each site relative to the fringe‐line in the interior of the forest. (c) At each site a temperature logger was deployed and six “sets” of tea bags were arranged around the logger, about 2 m apart. (d) Each “set” consisted of four tea bags (two green, two red). Tea bags were deployed in January 2022, half of which were retrieved after 3 months, alongside the temperature loggers, and the remainder after 12 months.

This study was initiated in the austral summer of 2022 in 30 estuaries along the east coast of the North Island of Aotearoa New Zealand (Figure 1a). To minimize climatic variability along this gradient, sampling sites were selected in a narrow latitudinal window spanning only 140 km from north to south (37°43′20.09″ S to 36°36′46.08″ S). The only mangrove species present was *Avicennia marina* subsp. *australasica*. Sites were selected along a land use gradient that ranged from 0% to > 80% combined agricultural and urban development in the catchments. We previously showed that this gradient was correlated with increased indicators of eutrophication (increased porewater nutrient concentrations and foliar δ^15^N and reduced oxygen reduction potential and pH) in catchments of higher human land use (Thomson et al. 2024).

A detailed description of sites and sampling procedures is given in Thomson et al. (2024). Briefly, each study site was located 15 m into the interior of the forest perpendicular to the seaward fringe line (Figure 1b). Following the method of the Tea Bag Index (TBI) outlined by Keuskamp et al. (2013), we buried two types of tea (Lipton green tea (EAN: 87 22700 05552 5) and Lipton rooibos (red) tea (EAN: 87 22700 18843 8)) in surface sediments of mangrove forests to assess the rate at which they decompose. At each site, one temperature logger and 12 green and red tea bags were buried approximately 5 cm below the sediment surface, in January 2022. Four tea bags (2 green and 2 red) were grouped together in a set (replicate) and buried about 0.2 m apart. Spacing between the 6 sets was about 2 m (Figure 1c). Six of each color tea bags (one from each set) and temperature loggers were retrieved during the first sampling period in April 2022, and the remaining six tea bags of each color were retrieved in April 2023. The tea bags had been incubated for a period of 3 months (92 days) and 12 months (372 days) (Figure 1d).

### 2.2 Decomposition Assays

We used standardized assays of labile (green tea) and recalcitrant (red tea) organic material. Green tea represents a more labile material source (representative of leaves and fine roots) and is expected to decompose faster, whereas the red tea is made up of more recalcitrant material with expected slower rates of decomposition, such as woody plant components (Keuskamp et al. 2013). As a modification to the original TBI, we incubated additional bags for 12 months, as some studies have found long‐term incubation data to be better related to environmental factors (Marley et al. 2019). A description of the methods used has been provided in the Supporting Information (Text S1). Briefly, initial and remaining dry weight of the tea was measured. We used this time series data of each tea type to calculate decomposition rate (*k*) by fitting a single‐term exponential decay model to the data at each site (*n* = 6 at each site), using a nonlinear least squares regression with the Levenberg–Marquardt algorithm for parameter estimation. The model was fitted using the nlsLM() command from the minpack.lm package *v1.2‐3* in *R* (Elzhov et al. 2023) and defined as:

(1) $$y=y_{0}e^{-\mathrm{kt}}$$

where *y* is the mass remaining at a time point, *y*_0_ is the initial mass at *t*_0_, *k* is the decomposition rate (mg day^−1^), and *t* is the time elapsed since the beginning of the incubation (in days). Henceforth, the decomposition rates (mg day^−1^) are expressed as *k*_green_ and *k*_red_ for green and red tea, respectively. To facilitate comparisons with studies following the TBI protocol, *k*_TBI_ and the stabilization factor (*S*_TBI_) were calculated (Keuskamp et al. 2013) and reported in the Supporting Information (Table S1).

### 2.3 Environmental Variables

All environmental variables were collected within a 2 m radius of the tea bags and replicates distributed spatially to capture the variability at each site. We therefore expect the variables to represent the average environments directly adjacent to the decomposition assays. Sediment temperature at each site, at 5 cm below the surface next to the decomposing tea bags, was continuously logged (sampling frequency 0.5 h, sampling resolution 0.1°C; EnvLogger T2.4, electricblue, Portugal) for the first 3 months (Jan–April 2022) of the 12‐month experiment. The site‐specific temperature data captured sources of variation including local topography, shading, moisture content, and local warming/cooling of water/air, and as such differences between sites were therefore expected, despite the narrow climatic range between sites. Although temperatures records were only collected for the first 3 months of decomposition, this can be considered as the “active” period, where most of the mass will be lost (Keuskamp et al. 2013; Trevathan‐Tackett et al. 2021). Furthermore, relative differences in temperature between sites were expected to persist over time. All other environmental variables were measured only once in mid‐April 2022. A detailed description of the sampling procedures and analytical methods for environmental variables is given in Thomson et al. (2024). Briefly, in situ measurements of pore water salinity, pH, and oxygen reduction potential (ORP) were taken from a dug‐out hole with a handheld multimeter probe (YSI ProQuatro, Xylem, Washington DC). Crab burrow density was counted from three randomly placed quadrats (0.25 m^2^). Burrow sizes were distinguished between large (> 2 cm) and small (< 2 cm) and their density was reported m^−2^. Samples for sediment parameters were collected using a 60 mL polyethylene syringe core (Ø 3 cm). At each site, nine cores of surface sediments (0–2 cm) were taken randomly near the buried tea bags. Three cores were then combined into one composite sample to account for small‐scale local variability resulting in three pooled samples. This collection method was repeated for the porewater samples. Leaves were sampled from three random trees in the sampling area. Three shoots with 10 or more apical green leaves were collected from each tree. All samples were kept in the dark and on ice and placed in a freezer after sampling was completed (after 3 h max). Porewater was extracted by centrifugation as soon as possible after sampling and the supernatant was filtered through 0.7 μm filters (GF/F Whatman) before being separated into nutrient and dissolved organic carbon (DOC) samples. Nutrient samples were frozen at −20°C while DOC samples were protected from light and cooled at 4°C until analysis.

Sediment‐related variables included grain size (mud content < 63 μm), water content, chlorophyll *a*, phaeopigment, total organic matter (TOM), total organic carbon (Sediment C), total nitrogen (Sediment N), sediment δ^13^C (carbon isotope ratio), and sediment δ^15^N (nitrogen isotope ratio). Porewater variables included nitrate and nitrite (NO_x_), ammonium (NH_4_^+^), phosphate (PO_4_^3−^), and dissolved organic carbon (DOC). Foliar variables included foliar total organic carbon (foliar C), foliar total nitrogen (foliar N), foliar δ^13^C, and foliar δ^15^N. A table of all environmental variables, abbreviations, and units used in the statistical analysis can be found in Table S2.

### 2.4 Statistics

Some explanatory variables (DOC, NO_x_, PO_4_, crab burrows (< 2 cm), and crab burrows (> 2 cm)) were log+1 transformed to eliminate the impact of a few high values, while others (TOM, mud content, water content, ratio chl *a*:phaeo, tree density, %Human) were logit‐transformed as suggested for proportionate data (Warton and Hui 2011). Three missing predictor values (variable: DOC from Tuapiro) and six missing response values (variable: green tea (after 12 months) from Manaia) were imputed using the missForest() command from the missForest *v1.5 package* in *R* (Stekhoven and Bühlmann 2012). To capture the daily temperature variation at each site, the temperature measured from each logger was separated by day, and each daily record was then aggregated into quantiles (Min, 5th, 10th, 25th, Median, 75th, 90th, 95th, Max). Individual linear models were fitted to determine which daily temperature quantile best explained decomposition rate to avoid overfitting with multiple collinear metrics. For both substrates the lower temperature quantiles (minimum to 25th quantile) explained more variance (Green tea: *R* ^2^ = 0.18; Red tea: *R* ^2^ = 0.35) in the data compared to the higher quantiles (median to maximum; Green tea: *R* ^2^ = 0.01; Red tea: *R* ^2^ = 0.15; Figure S1). Therefore, we used the 25th quantile of daily temperature fluctuation (from here on called T_Q25_) as the temperature predictor variable in the analyses.

Overall, 32 environmental variables were compiled (Table S2). To avoid overfitting, which can have negative effects on the model performance and interpretability through collinear variables (Dormann et al. 2013), the Pearson's correlations of the environmental matrix were assessed. From variable pairs that exceeded a correlation score of > 0.75, one variable was removed prior to the analysis. This is commonly done to reduce high dimensional datasets, especially if the variables are ecologically linked or they represent similar conditions or processes. For example, water content was removed due to its high inverse correlation with TOM, as less dense sediment can hold more water in the interstitial spaces. The variance inflation factors (VIF) of each variable were subsequently calculated to confirm a low remaining level of collinearity (VIF < 10). It is important to note that the excluded variables may remain important drivers of decomposition in mangrove sediments, and that they were considered when assessing the results of the statistical analysis.

To identify the individually most influential environmental variables on *k*_green_ and *k*_red_, the marginal effects of each predictor variable on the various response variables were assessed using the add1() command from the stats package *v 4.2.1* in R. This was done after checking the influence of the factor “Site” as a random variable using a linear mixed‐effects model (by the variance term in the summary output) which had no effect. The multiple R^2^ values of the summary output were recorded as a measure of the strength of the marginal relationship between the dependent and explanatory variables.

To assess the combined effects of the environmental variables on decomposition, separate Random Forest (RF) regression models were constructed, with the environmental variables presented above as independent predictors (Breiman 2001). The RF models were built using the randomForest package *v4.7‐1.1* in R (Liaw and Wiener 2002) and tuned to select optimal values for model parameters *mtry* (the number of variables used in each tree node), *maxnodes* (the maximum number of terminal nodes), and *ntree* (the number of trees to grow). Bootstrapping (100 iterations) was used to incorporate uncertainty into the model performance and variable importance estimates. Model performance was assessed using the % variance explained (%var.; *R* ^2^ values estimated from the internal out‐of‐bag error), and the mean squared residuals (MSE). Variable importance was calculated from the “%IncMSE” value and expressed as relative importance of that variable in respect to all other variables included in the model.

Partial dependence plots (PDP) of the random forest were utilized to establish relationships between selected variables and decomposition metrics while eliminating confounding effects of other variables. To investigate how the nutrient enrichment in combination with temperature changes interacted with decomposition, bi‐variable partial dependence plots were created from selected variables. Partial dependence was calculated using the R package “pdp” *v. 0.8.1* (Greenwell 2017), and uncertainty estimates were calculated using bootstrap sampling of the model predictions. All data analyses were done using R (R Core Team 2021).

## 3 Results

### 3.1 Decomposition Rates

**FIGURE 2:**
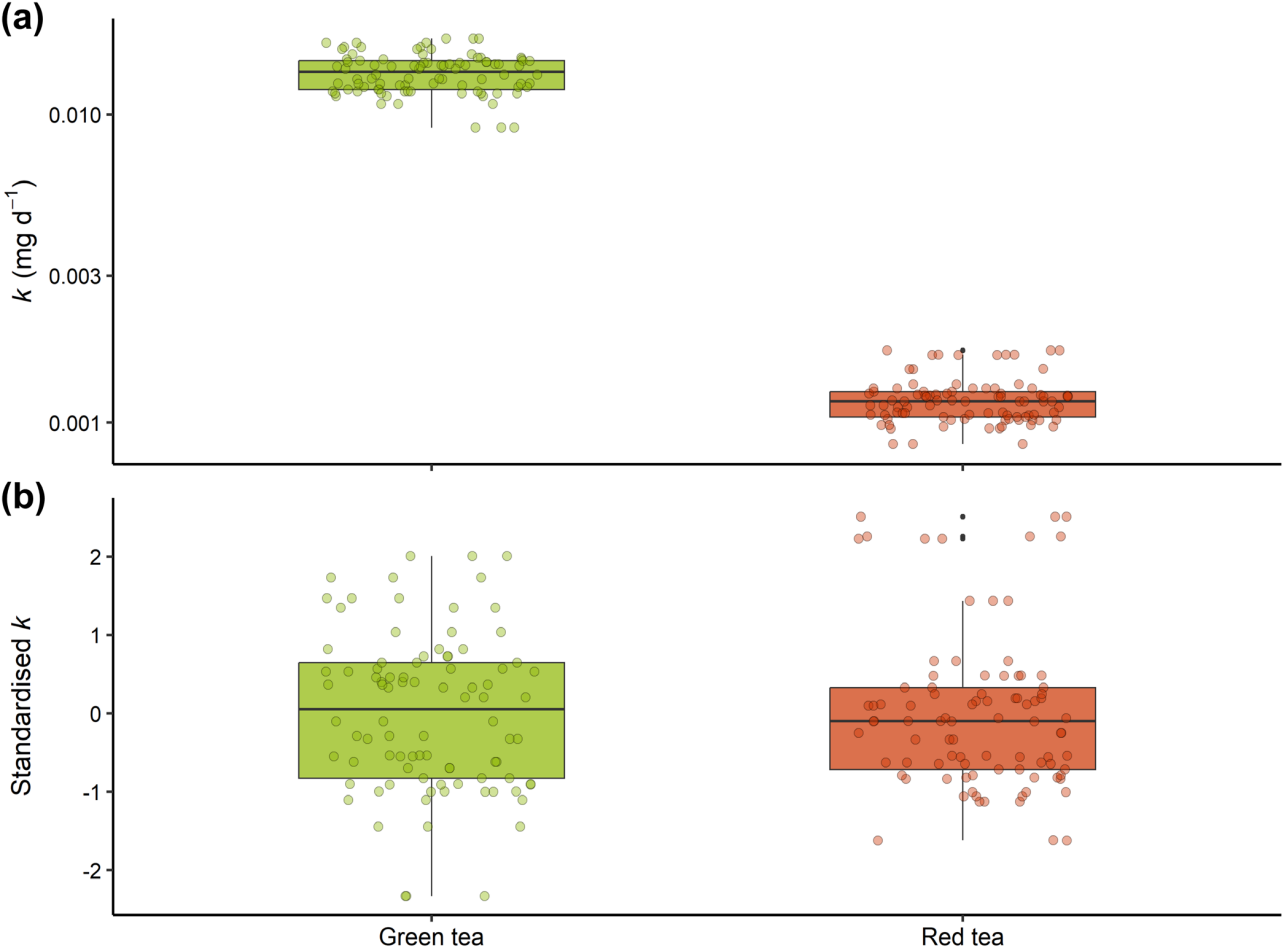
Range and variance of decomposition rates of green tea, and red tea in temperate mangrove forests. (a) Decomposition rate as calculated using a single‐term exponential decay function from the remaining dry weight of tea over three time‐points. (b) Normalized (standardized and centralized) values of decomposition. Data from all 30 sites with three replicates at each site are presented here as independent points (*n* = 90). The boxplot centerline indicates the median, the box indicates the 25th and 75th percentiles (IQR) and the whiskers indicate the smallest and largest values within ±1.5 × IQR. Black points outside the whiskers indicate values that lie beyond the ±1.5 × IQR. Underlying data are shown as colored points.

The more labile green tea lost 73% ± 4% (mean ± standard deviation) of its initial mass over the first 3 months of incubation, while the refractory red tea lost only 25% ± 4%. After 12 months, the mass loss had increased to 81% ± 3% and 36% ± 5% for the green and red tea, respectively (Table S1). The differences in mass‐loss were reflected in the decomposition rates, with *k*_green_ (6.7 ± 1.0 mg day^−1^) being an order of magnitude greater than *k*_red_ (0.6 ± 0.1 mg day^−1^). While *k* varied by an order of magnitude between organic matter source (Figure 2a), the standardized values indicated a similar level of variance (Figure 2b).

### 3.2 Influence of Environmental Controls Depends on Substrate Recalcitrance

**FIGURE 3:**
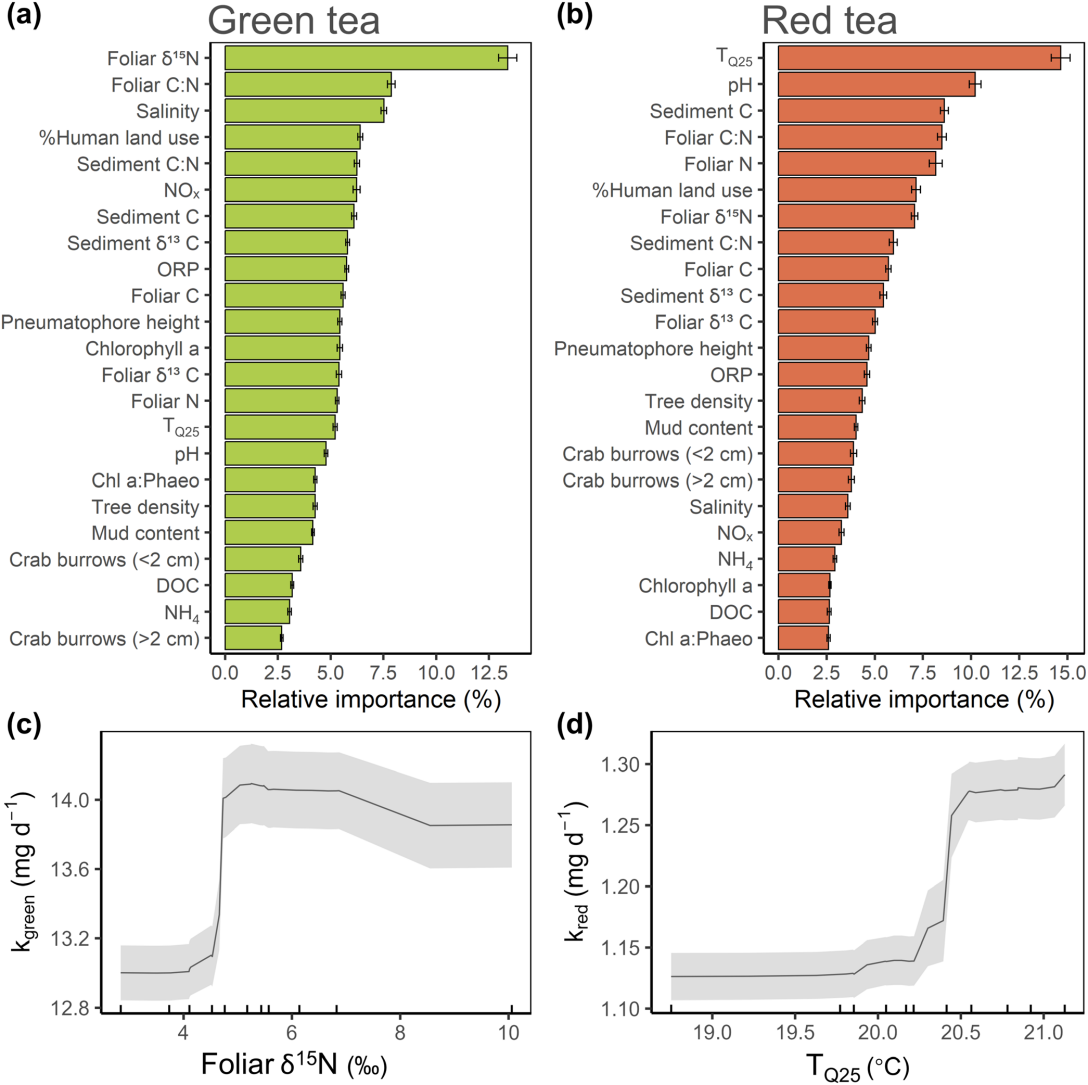
Environmental drivers of decomposition in temperate mangrove forests. Importance of the predictor variables for labile green tea (a) and recalcitrant red tea (b) decomposition as determined by random forest analysis (*n* = 90). Explanatory variables are arranged in order of their relative importance to the model outcome. Variable importance was based on the improvement of the mean squared residuals (MSR) and displayed as relative to the variance explained by all variables. Bar charts indicate the average relative importance of each variable and error bars indicate one standard deviation estimated from 100 bootstrap iterations. Partial dependence plots of the highest ranked variable for each substrate are shown in (c) and (d). The black line indicates the mean and the ribbon the standard deviation of the 100 bootstrap iterations. C = carbon; Chl a:phaeo = chlorophyll a to phaeophytin ratio; DOC = dissolved organic carbon; N = nitrogen; NH_4_ = ammonium concentration; NO_x_ = nitrate/nitrite concentration; ORP = oxygen reduction potential; T_Q25_ = daily temperature (25th percentile).

The model fit of the random forest models was considered good for both *k*_green_ and *k*_red_ (Table 1), but the recalcitrance of the substrate materials (i.e., difference between *k*_green_ and *k*_red_) altered relationships with environmental variables. All explanatory variables used (*n* = 23; Table S2) had relative importance values of > 2.5% in both models, and no single variable contributed more than 15% to the explanatory power of each model (Figure 3a,b; Tables S4 and S5). This highlights the complexity and multitude of factors influencing organic matter decomposition in mangrove forests at landscape scales. From the marginal predictors, *k*_green_ was controlled by several highly influential variables of similar strength (specifically: porewater nitrate/nitrite concentrations (NO_x_), T_Q25_, oxygen reduction potential (ORP)), while *k*_red_ was mainly influenced by the most important single predictor, T_Q25_ (Table S3).

**TABLE 1:** Performance of the random forest models. Total variance explained and mean squared error from the 23 environmental predictor variables. Means ± standard deviations were calculated using 100 bootstrap iterations.

Decomposition constant	%‐var explained	Mean squared error (MSE)
*k*_green_	90.6 ± 8.2	0.86 ± 0.65
*k*_red_	92.0 ± 7.8	0.008 ± 0.007

The set of environmental variables that best explained decomposition from the random forest model was specific to each organic matter substrate. Furthermore, many of the relationships between predictor variables and organic matter decomposition were nonlinear, revealing thresholds of environmental factors beyond which decomposition increased substantially (e.g., Figure 3c,d).

**FIGURE 4:**
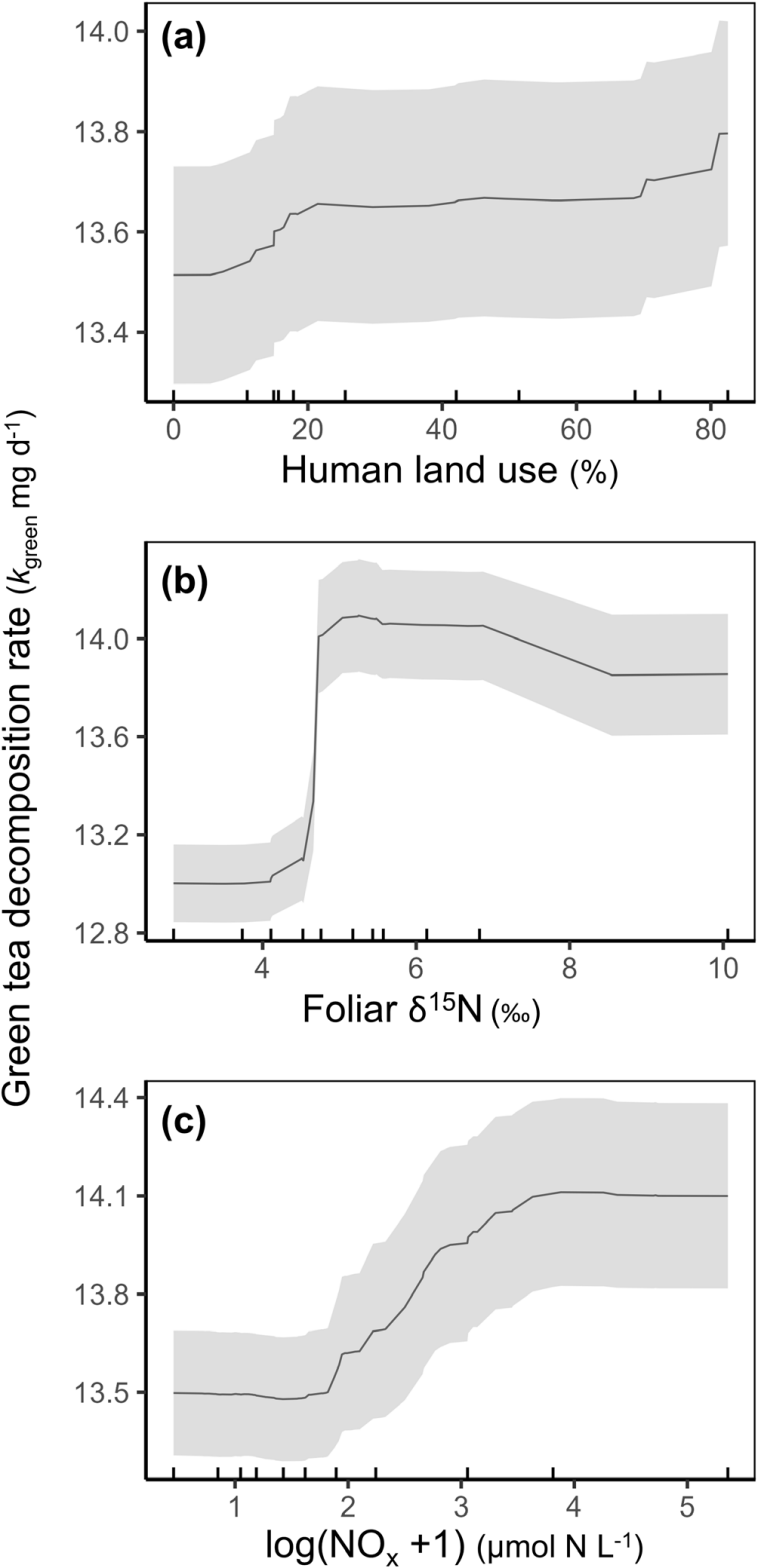
Human land use induced effects on decomposition of labile organic matter (green tea) in temperate mangrove forests. Partial dependence plots (PDP) of selected variables returned from the random forest model (*n* = 90) of (a) %Human land use in catchments, (b) foliar δ^15^N, and (c) nitrate/nitrite concentrations in the porewater. The black centerline indicates the mean PDP estimate, and the grey ribbon shows the standard deviation of 100 bootstrap iterations.

For the decomposition of labile substrate (*k*_green_), foliar δ^15^N was the best predictor (13.3% ± 4.2%; relative importance of predictor variable; bootstrap mean ± standard deviation), and almost twice as important as each of the next most important variables: foliar C:N (7.9% ± 1.7%), salinity (7.5% ± 1.4%), %Human land use (6.6% ± 1.1%), sediment C:N (6.4% ± 1.1%), and NO_x_ (6.2% ± 1.7%). Foliar δ^15^N, %Human land use, and NO_x_ were positively correlated with *k*_green_ (Figure 4). Contrary to expectations, foliar C:N ratio was also positively correlated with *k*_green_ (Figure S3). Salinity and sediment C:N ratio were both negatively correlated with *k*_green_ (Figure S3). For the more recalcitrant material (*k*_red_), T_Q25_ was the most important predictor, with a relative importance of 14.7% ± 4.9%, followed by pH (10.2% ± 3.1%), sediment C (8.6% ± 2.0%), Foliar C:N (8.5% ± 2.3%), Foliar N (8.2% ± 3.3%), %Human land use (7.1% ± 2.3%), and foliar δ^15^N (7.1% ± 1.6%) (Figure 3b; Table S4). There was a threshold at about 20.4°C, where *k*_red_ increased sharply by 13% (from 1.13 to 1.28 mg day^−1^) (Figure 3d). Similarly, a pH increase from 7.1 to 7.2 was related to a *k*_red_ increase of 7% (Figure S4). Sediment C and foliar C were both negatively correlated with *k*_red_, while foliar C:N ratio, %Human land use, and foliar δ^15^N all were positively correlated with *k*_red_. Sediment texture variables (i.e., mud content) ranked low in both models, suggesting a low explanatory power of these parameters on organic matter decomposition (Figure 3a,b).

### 3.3 Potential Links Between Human Land Use and Decomposition in Mangrove Forests

The proportion of land in the catchment utilized by humans for either urban or agricultural purposes (%Human land use) was a good predictor of decomposition across both substrate types (6.6% ± 1.1% for *k*_green_, 7.1% ± 2.3% for *k*_red_; Figure 3a,b; Tables S4 and S5). The partial dependence plot indicated a positive correlation between %Human land use and decomposition of green tea, which increased continuously with human land use (Figure 4a). No clear correlation pattern was discernible between human land use and red tea (Figure S4). Since green tea was the most susceptible to human‐derived influences, we focused our attention on the relationships between environmental factors and *k*_green_.

**FIGURE 5:**
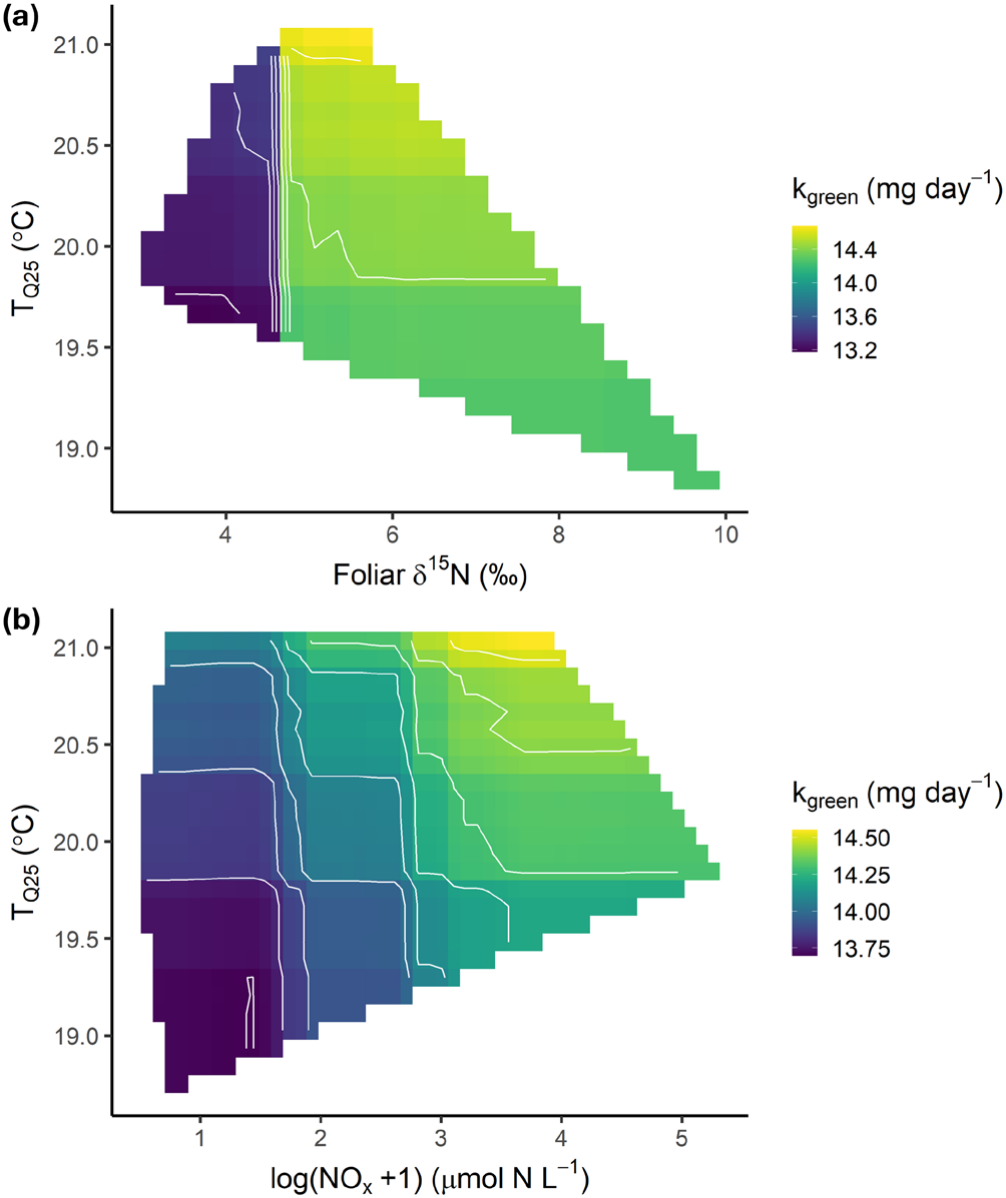
Interactive effects of temperature and eutrophication on decomposition rates of labile organic matter. Two variable partial dependence plots of high‐ranking indicators of eutrophication with T_Q25_ (daily temperature (25th percentile)) on the decomposition of labile organic matter (green tea), determined by random forest analysis (*n* = 90). (a) Foliar δ^15^N and T_Q25_, (b) porewater nitrate/nitrite concentrations and T_Q25_. The estimates are means created by 100 bootstraps.

Mangrove foliar δ^15^N and porewater NO_x_ concentrations were high‐ranking variables in predicting decomposition of labile organic matter (green tea) in this study (Figure 3a). Both variables are indicators of nutrient enrichment and have previously been shown to be positively correlated with human land use (Thomson et al. 2024). Values of δ^15^N above 4‰ and porewater NO_x_ concentrations of about 4 μmol N L^−1^ and higher were related to higher decomposition rates (Figure 4b,c). At NO_x_ concentrations of about 32 μmol N L^−1^, this relationship seemed to reach a plateau, after which decomposition did not increase with higher NO_x_; however, low numbers of samples with high NO_x_ concentrations may have influenced this relationship. Bi‐variable partial dependence plots were investigated to assess the effects of these two variables in relationship with temperature. T_Q25_ was the second‐best predictor of *k*_green_ from the marginal test but ranked 11th in variable importance in the random forest model (Figure 3a; Tables S3 and S4). This disconnect indicates possible interaction effects of T_Q25_ with other predictor variables. Notably, above the thresholds of 4.5‰ (foliar δ^15^N) and 4 μmol N L^−1^ (NO_x_), the temperature sensitivity of green tea decomposition increased strongly (i.e., the decomposition rate was increased by lower temperature thresholds when nutrient indicators were high), suggesting an accelerated loss of labile organic material to warming in a eutrophied system (Figure 5a,b). These bi‐variable relationships were similarly tested for *k*_red_ (between T_Q25_ and pH, Foliar δ^15^N, and %Human land use); however, temperature dominated each of these interactions (Figure S6).

## 4 Discussion

The results presented here establish a link between land‐based stressors and the accelerated decomposition of labile organic matter, suggesting that anthropogenic nutrient enrichment negatively impacts carbon sequestration rates in temperate mangrove forests. Furthermore, the accelerating effects of eutrophication on decomposition increase the sensitivity of this process to temperature, indicating a synergistic weakening of the carbon storage potential through combined local and global stressors. It is important to note, however, that these same drivers could also affect carbon inputs into the ecosystem, leading to uncertainty about their overall effect on carbon sequestration in mangrove forests. This study provides evidence that environmental drivers of organic matter decomposition in temperate mangrove forests differ with varying recalcitrance of the decomposing material. While the labile substrate (green tea) was strongly affected by several eutrophication indicators, decomposition of the recalcitrant substrate (red tea) was mainly influenced by fluctuations in lower daily temperatures.

Integrated measures of environmental conditions over time (daily temperature, Foliar δ^15^N) were identified as important predictors of green and red tea decomposition. This relationship is likely due to the variance introduced by using single measurements in time (such as porewater nutrient concentrations) as representative of a longer time series with intra‐ and interannual variation in that metric, making them less suited for tracking temporal processes. Temperature was measured for the first 3 months of the 12‐month incubation period. However, most mass was lost during this period (96% ± 30% and 69% ± 12% of total mass lost after 12 months, for green tea and red tea, respectively), corroborating this to be the active period of decomposition (Trevathan‐Tackett et al. 2021). The three‐month data was hence used as an indicator of the thermal condition at each site, which we expect to be representative of site‐to‐site variations that persist over time. The use of standardized materials to test for decomposition at all sites enables the results to be unequivocally attributed to environmental effects on the organic matter substrate rather than to innate properties of local litter. Moreover, these relationships were reproduced across a wide range of estuaries, giving confidence about the wider applicability of our results.

### 4.1 Eutrophication Accelerates Labile Organic Matter Decomposition

The decomposition of labile organic matter (green tea) was strongly influenced by a set of variables that relate to enriched nutrient conditions reflecting human land use in the catchments (δ^15^N, NO_x_, and the ratio of C:N (in leaves and sediments)). Foliar δ^15^N values are considered good proxies for anthropogenic nutrient enrichment as they can provide an integrated, landscape‐scale perspective of nitrogen cycling (Fry et al. 2003). In mangrove forests, δ^15^N has been shown to correlate with anthropogenic land use and exposure to elevated nutrient concentrations (Gritcan et al. 2016; Tanu et al. 2020; Thomson et al. 2024). The spatiotemporally integrated nature of δ^15^N as a proxy for high nutrient loading may explain why it was a better predictor for *k*_green_ than point measurements of porewater or sediment concentrations of nutrients, which are transient indicators of environmental conditions. The variables controlling *k*_green_ imply that the more labile fraction of organic matter is vulnerable to the effects of catchment human land use on coastal ecosystems. The threshold of about 4.5‰ of δ^15^N, indicated by the partial dependence plot (Figure 4b), after which a nonlinear increase in decomposition occurs, is at the upper range of natural values for a variety of tree leaves (−8‰ to 3‰) (Peterson and Fry 1987) and specifically mangrove leaves (−11‰ to 3.8‰) (Costanzo et al. 2001; Fogel et al. 2008; Reis et al. 2017). Moderate to high NO_x_ concentrations (> 4 μmol N L^−1^) also accelerated the decomposition of green tea, supporting this hypothesis. Hence, the decomposition rate of labile organic matter was faster in mangrove forests that showed evidence of exposure to increased nutrient loading by anthropogenic influences.

**FIGURE 6:**
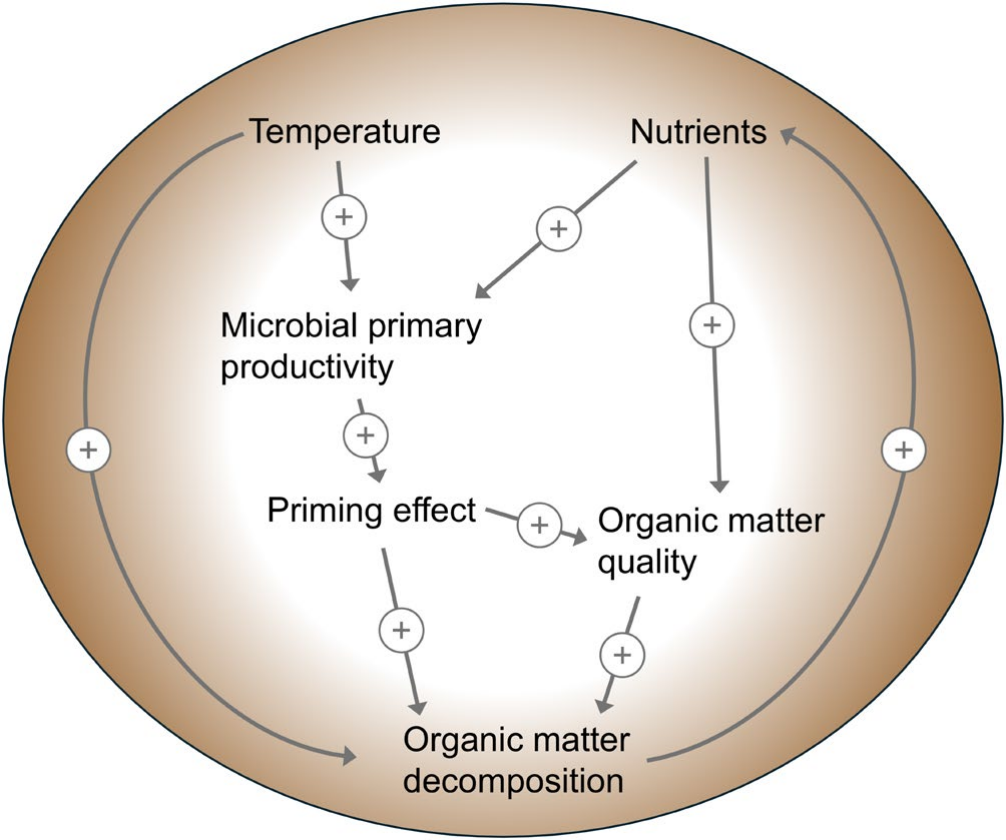
Conceptual representation of important pathways controlling the rate of organic matter decomposition in mangrove sediments. The priming pathway through microbial primary production (microalgae) sets it apart from most terrestrial systems, potentially making decomposition more vulnerable to nutrient inputs in aquatic settings. Temperature affects decomposition directly as well as through the microbial priming effect. Nutrients promote microbial priming as well as the overall quality of the organic matter through increased leaf growth, lower C/N ratio of plant tissues, and reduced nutrient retention. The dual effect of temperature and nutrient enrichment on the microbial priming effect may explain the synergism of these two stressors on the decomposition rate. Accelerated organic matter decomposition in turn promotes the remineralization of nutrients potentially leading to a positive feedback in enriched systems.

Increased concentrations of nitrogen in various forms have been linked to higher rates of carbon efflux (CO_2_ and CH_4_) from mangrove sediments (Barroso et al. 2022). While CO_2_ efflux is commonly used as a proxy for decomposition processes, a direct relationship between nutrient concentrations and decomposition has not yet been observed in mangrove forests (Jessen et al. 2021). We propose here that the inhibitory effect of nitrogen enrichment on decomposition often observed in terrestrial systems, does not apply in habitats where primary production by microbial autotrophs also occurs. Instead, increased labile carbon sources produced by these autotrophs in response to inorganic nutrient delivery alleviate carbon limitation and promote organic matter decomposition in sediments (Zhu et al. 2024) (Figure 6). Furthermore, the indirect effect of eutrophication on decomposition via increasing litter quality may also affect decomposition rates (Fog 1988; Jessen et al. 2021; Keuskamp et al. 2015) (Figure 6). Here, *k*_green_ was positively correlated with mangrove foliar C:N ratio (Figure S3), contrary to the expectation that lower C:N ratios benefit decomposition. However, the sampled mangrove leaves were not representative of the tea litter, or of the senescent leaves of the forest. High nutrient concentrations in the porewater may be a result of organic matter decomposition through the release and remineralization of nutrients from the litter (Keuskamp et al. 2015; Mamidala et al. 2023; Zhang et al. 2018). However, the high concentrations of nutrients in the porewater at some sites (particularly in urban settings) likely point to an external nutrient source. In the case of coastal eutrophication, a combination of direct and indirect effects on decomposition may create a positive feedback loop (Figure 6) that leads to a permanently enriched system in which carbon sequestration is substantially reduced (Branoff 2017).

### 4.2 Decomposition of Recalcitrant Material is Driven by Temperature

The decomposition of recalcitrant organic matter (red tea) was primarily controlled by sediment temperature (Figure 3b,d). Temperature is well known to be an important driver of organic matter loss from soil and sedimentary carbon stocks (García‐Palacios et al. 2021; Nissan et al. 2023; Trumbore and Czimczik 2008; von Lützow and Kögel‐Knabner 2009). Microbial activity and enzymatic reactions are temperature‐dependent, and a conventionally agreed doubling of sediment OM decomposition with every 10°C rise in temperature (Q_10_) has commonly been applied in the literature (Davidson and Janssens 2006). Therefore, the positive relationship between the decomposition rate of OM and temperature in mangrove sediments is not surprising. It has been theoretically and empirically shown that temperature sensitivity of organic matter decomposition increases with increasing recalcitrance of the substrate, which can be explained by the difference in activation energy required to start decomposition processes on materials of varying complexity (Craine et al. 2010; Davidson and Janssens 2006; Fierer et al. 2005; Jia et al. 2020; Qin et al. 2019). The fact that the more recalcitrant substrate used in this study (red tea) showed the strongest relationship with temperature corroborated this theory. It furthermore highlights the importance of considering different pools of organic matter to accurately predict changes in decomposition with climate or human impacts (Davidson and Janssens 2006).

Interestingly, the lower percentiles of daily temperature were better at explaining decomposition rates compared to the average or the upper daily temperature percentiles (Figure S1). This suggests that changes in the lower temperature threshold are more likely to be driving decomposition processes in temperate mangrove sediments. Therefore, increases in the daily temperature minima need to be considered when addressing climate change stressors in coastal ecosystems, not just shifting temperature maxima. For example, increasing night‐time temperatures could lead to dramatic increases in OM decomposition, while day‐time decomposition rates are less affected. How future changes of temperature extremes affect organic matter inputs remains unclear; however, variations in the response could potentially decouple organic matter inputs from decomposition processes under changing temperature conditions. This finding has implications for a need to consider not simply extreme heat wave events but also lower temperatures and general warming trends that coastal marine environments are experiencing globally. For example, the decomposition rate of red tea increased by about 13% (from ~1.13 to 1.28 mg day^−1^) when T_Q25_ surpasses 20.4°C indicating a potential vulnerability threshold of sedimentary organic matter at that temperature. These thresholds are to be considered with care however, as the temperature was only measured for 3 months in summer and continuous seasonal records may affect this estimate. This relationship between temperature minima as driving factor of decomposition needs to be studied in more detail.

While %Human land use was a good predictor for *k*_red_, its association with the response variable remains uncertain, as the partial dependence plot did not show a clear directional relationship (Figure S4). This may be due to interactions of %Human land use with other predictors in the measured variables; however, no clear interactive relationships were found due to the dominance of temperature as a predictor (Figure S6).

### 4.3 Does Eutrophication Exacerbate the Effects of Warming on Decomposition?

The interaction effect between temperature and nutrient indicators on the labile organic matter substrate (green tea) is particularly noteworthy (Figure 5). These results suggest a reduced temperature threshold for decomposition in eutrophied systems, which has implications for local human impacts on carbon sequestration in a warming world. While human‐induced eutrophication is already impairing the ability of coastal wetlands to reach their full carbon storage potential (Ouyang et al. 2023), this relationship may deteriorate nonlinearly with rising global temperatures. It is important to note that this hypothesis is based on limited data, especially in the extreme values, and requires further investigation. Nonetheless, existing literature supports the notion that nutrient additions increase the temperature vulnerability of sediment organic carbon and leaf litter (Knorr et al. 2024; Yang et al. 2024). This effect is generally attributed to shifts in bacterial and/or fungal communities and their enzymatic activities, combined with changes in litter‐, microbial‐, and environmental stoichiometry (Fernandes et al. 2014; Ferreira and Chauvet 2011; Knorr et al. 2024). We propose here another pathway in aquatic habitats, where temperature and eutrophication in combination may synergistically affect microbial priming, leading to the observed increased vulnerability of organic matter in mangrove sediments (Tian et al. 2021) (Figure 6).

## 5 Conclusions

This study provides regional‐scale empirical evidence for the loss of labile organic carbon due to coastal eutrophication and highlights a weakening of the temperature‐related inhibition of OM decomposition at sites exposed to high nutrient inputs. While this study used a standardized allochthonous substrate, preventing direct calculation of representative carbon loss rates relative to fixation rates, the findings underscore the need for further research on site‐specific litter characteristics and their influence on decomposition. Moreover, the effects of environmental drivers on carbon inputs and losses need to be assessed simultaneously, to understand their overall effects on carbon sequestration. For example, evidence suggests that increased eutrophication can lead to higher rates of carbon fixation by primary producers, possibly offsetting higher losses through decomposition (Hayes et al. 2017; Janssens et al. 2010; Keuskamp et al. 2015). How these interactions evolve over longer time scales remains unclear. Nutrient‐rich systems tend to produce more, and higher quality litter, leading to self‐fertilization with direct and indirect effects on decomposition (Fog 1988; Mamidala et al. 2023). The high carbon sequestration potential that mangrove ecosystems provide may partly be due to their ability to protect a fraction of the labile OM from decomposition, which is incorporated into the sediment matrix or the microbial loop, to last for centuries (von Lützow et al. 2006). Effective management of terrestrial runoff could enhance ecosystem productivity while minimizing the loss of the dynamic and vulnerable fraction of organic matter from sediments.

## Author Contributions

**Timothy Thomson:** conceptualization, data curation, formal analysis, funding acquisition, investigation, methodology, project administration, visualization, writing – original draft, writing – review and editing. **Conrad A. Pilditch:** conceptualization, methodology, supervision, writing – original draft, writing – review and editing. **Marco Fusi:** conceptualization, formal analysis, methodology, supervision, writing – review and editing. **Natalie Prinz:** investigation, visualization, writing – review and editing. **Carolyn J. Lundquist:** funding acquisition, methodology, writing – review and editing. **Joanne I. Ellis:** conceptualization, funding acquisition, methodology, supervision, writing – original draft, writing – review and editing.

## Conflicts of Interest

The authors declare no conflicts of interest.

## Supporting information


Data S1.

## Data Availability

The data that support the findings of this study are openly available via figshare at https://doi.org/10.6084/m9.figshare.27895638 and https://doi.org/10.6084/m9.figshare.28339916.
