# Supplementary material for: Vulnerability of Labile Organic Matter to Eutrophication and Warming in Temperate Mangrove Ecosystems
Source: Glob Chang Biol. 2025 Feb 14;31(2):e70087. doi: 10.1111/gcb.70087 (PMC11826976; doi:10.1111/gcb.70087)
Supplement: Supplementary file 1 — Data S1. [file GCB-31-e70087-s001.docx]

Vulnerability of labile organic matter to eutrophication and warming in temperate mangrove ecosystems

Supplementary materials

Timothy Thomson^a^*, Pilditch, C. A.^a,b^, Fusi, M.^c^, Prinz, N.^a^, Lundquist, C. J.^b,d^, Ellis, J. I.^a^

^a^School of Science, University of Waikato, Tauranga, New Zealand

^b^School of Environment, University of Auckland, Science Centre, 23 Symonds Street, Auckland 1010, New Zealand

^c^Dove Marine Laboratory School of Natural and Environmental Sciences, Newcastle University, Newcastle NE1 7RU, UK

^d^National Institute of Water & Atmospheric Research, PO Box 11115, Hamilton 3215, New Zealand

* Corresponding author.

Email address: [timi.thomson@gmail.com](mailto:timi.thomson@gmail.com)

Postal address: University of Waikato, Coastal Marine Field Station, Unit 3/4 58 Cross Road, Sulphur Point, Tauranga 3110, New Zealand

**Contents**

**Supplementary Text 1: Tea bag index protocol** A detailed description of the decomposition assays used in this study

**Fig. S1** The coefficient of determination (R^2^) of different temperature quantiles on the degradation rate green (k_green_) and red (k_red_) tea. Individual linear models were fitted to each quantile to establish the coefficient of determination of the relationship.

**Fig. S2** Correlation matrix of all environmental variables.

**Table S1** Summary table of different degradation metrics. The first two columns were used as response variables for green and red tea decomposition, respectively. The decomposition rate (k_TBI_) and stabilization factor (S_TBI_) were calculated using the methodology given in Keuskamp et al. (2013) for the Tea bag index studies and is listed here to facilitate comparisons with studies using the same methodology. Information on sites can be found in Thomson et al. (2024). Values printed in red were imputed by the random forest algorithm.

**Table S2** Measured environmental variable. Those shaded in grey were removed prior to the analysis to avoid collinearity and the correlated variables are shown in the last column. The remaining 23 variables were carried forward into the statistical analysis. Notations used in the text as well as the units are given.

**Table S3** Marginal effects of the ten most important individual predictors of the degradation of green (k_green_) and red (k_red_) tea.

**Table S4** Relative importance of predictor variables controlling green tea decomposition (k_green_) in temperate mangrove forests as calculated from the random forest model. Variables are ranked by importance within the model. Mean and standard deviation were calculated using 100 bootstraps.

**Table S5** Relative importance of predictor variables controlling red tea decomposition (k_red_) in temperate mangrove forests as calculated from the random forest model. Variables are ranked by importance within the model. Mean and standard deviation were calculated using 100 bootstraps.

**Fig. S3** Partial dependence plots of all variables’ relationship with degradation rate of green tea (k_green_) ordered by relative importance to the model performance. Black lines are means and ribbons represent the standard deviation from 100 bootstrap iterations.

**Fig. S4** Partial dependence plots of all variables’ relationship with degradation rate of red tea (k_red_) ordered by relative importance to the model performance. Black lines are means and ribbons represent the standard deviation from 100 bootstrap iterations.

**Fig. S5** Human land use induced effects on degradation of labile organic matter (green tea) in temperate mangrove forests. Two-variable partial dependence plots of daily temperature (25^th^ quantile) with **A** foliar C:N, **B** salinity, **C** sediment C:N, and **D** sediment C.

**Fig S6** Human land use induced effects on degradation of labile organic matter (green tea) in temperate mangrove forests. Two-variable partial dependence plots of daily temperature (25^th^ quantile) with **A** foliar C:N, **B** salinity, **C** sediment C:N, and **D** sediment C.

**Supplementary Text 1: Tea bag index protocol**

Following the method outlined by^1^, we buried two types of tea (Lipton green tea (EAN:87 22700 05552 5) and Lipton rooibos tea (EAN:87 22700 18843 8)) in mangrove sediments to assess the rate at which they decompose. Green tea represents a more labile material source, (representative of leaves and fine roots) and is expected to decompose faster, whereas the rooibos tea (from here on called ‘red tea’) is made up of more recalcitrant material with expected slower rates of decomposition, such as woody plant components^1^. The tea was contained in the original tea bags to allow for water flow and microbial access but restrict consumption by meio- and macrofauna. Before burial, each tea bag (tea, bag, string, and tag) was dried to a constant weight at 60 °C and then weighed. An average weight of each empty bag with string and tag was established empirically by weighing ten green tea bag and ten red tea bags. Differences between empty bags from green and red tea were tested using ANOVA (p-value = 0.82) and used to calculate a single average value seeing that there was no statistical difference between the weights of the bags. The variation around the mean from all bags was about 1.7%. The difference between the total weight of each bag and the average weight of an empty bag provided the initial weight of the tea in each bag. Once retrieved, the tea bags were washed in clean water and dried to constant weight at 60 °C. The dried tea was extracted from the bag and weighed to obtain the remaining weight after incubation. The difference between the initial weight of each tea bag and the remaining weight after incubation was the amount of tea lost during incubation. As a modification to the original TBI, we incubated additional bags for 12 months, as some studies have found long-term incubation data to be better related to environmental factors^2^. We used the time series data of each tea type to calculate decomposition rates by fitting a single-term exponential decay model to the data at each site, using a nonlinear least squares regression with the Levenberg-Marquardt algorithm as the measure of estimation^3^. The model was fitted using the nlsLM() command from the minpack.lm package *v1.2-3*^4^ and defined as:

$y= y_{0}e^{-kt}$ Eq. (2)

where *y* is the percent mass remaining at a time point, *y*_0_ is the initial mass at *t*_0_, and *t* is the time elapsed since the beginning of the incubation (in days). The decomposition rate was calculated from the red tea only, as there was not much change between the remaining mass of the green tea at 3 months compared to that after 12 months.

Calculations for decomposition rate constant (k) and stabilization factor (S) were performed following the TBI-protocol from^1^:

$W_{r}\left( t \right)= a_{r}*e^{-kt}+\left( 1-a_{r} \right)$ Eq. (3)

$S=1- \frac{a_{g}}{H_{g}}$*,* Eq. (4)

$a_{r}= H_{r}\left( 1-S \right)$*.* Eq. (5)

Where W_r_(t) describes the substrate weight of red tea after incubation time (t in days), a_r_ is the labile and 1 - a_r_ is the recalcitrant fraction of the substrate, and k is the decomposition rate constant. S describes the stabilization factor, a_g_ is the decomposable fraction of green tea (based on the mass loss during incubation), and H_g_ is the hydrolysable fraction of green tea. The decomposable fraction of red tea was calculated using Eqs. (4), based on its hydrolysable fraction (H_r_) and stabilization factor (S). With W_r_(t) and a_r_ known, k is calculated using Eq. (2). The decomposition rate calculated from the tea bag index was tested but yielded no relationship with any of the predictor variables, so it is not reported on in the manuscript. This is consistent with several studies that found the decomposition rate calculated from the tea bag index to be a very poor indicator of environmental variability^5,6^.

To enable the results from this study to be compared with other TBI studies, the decomposition rate and stabilisation factor are presented in Table S4.

Table S1: Summary table of different degradation metrics. The first two columns were used as response variables for green and red tea decomposition, respectively. The decomposition rate (k_TBI_) and stabilization factor (S_TBI_) were calculated using the methodology given in Keuskamp et al. (2013) for the Tea bag index studies and is listed here to facilitate comparisons with studies using the same methodology. Information on sites can be found in Thomson et al. (2024). Values printed in red were imputed by the random forest algorithm.

| **Site ID** | **Green tea decomposition rate (k_green_)** | **Red tea decomposition rate (k_red_)** | **Green tea (% loss after 3 months)** | **Green tea (% loss after 12 months)** | **Red tea (% loss after 3 months)** | **Red tea (% loss after 12 months)** | **Decomposition rate (k_TBI_)** | **Stabilization factor (S_TBI_)** |
| --- | --- | --- | --- | --- | --- | --- | --- | --- |
| ATH | 0.012 | 0.0010 | 70.28 ± 0.59 | 78.58 ± 1.66 | 24.05 ± 2.16 | 33.41 ± 1.33 | 0.0083 ± 0.0015 | 0.16 ± 0.03 |
| BOW | 0.017 | 0.0011 | 78.10 ± 1.40 | 82.71 ± 0.24 | 26.66 ± 0.90 | 36.98 ± 0.88 | 0.0087 ± 0.0006 | 0.07 ± 0.02 |
| COR | 0.016 | 0.0012 | 77.39 ± 1.54 | 81.02 ± 1.48 | 28.99 ± 2.14 | 35.48 ± 0.72 | 0.0100 ± 0.0010 | 0.08 ± 0.02 |
| HEI | 0.011 | 0.0013 | 67.62 ± 1.21 | 76.52 ± 1.88 | 16.35 ± 3.92 | 37.20 ± 2.80 | 0.0057 ± 0.0015 | 0.20 ± 0.01 |
| KAT | 0.015 | 0.0013 | 74.56 ± 3.03 | 84.06 ± 1.05 | 28.55 ± 5.42 | 37.42 ± 3.00 | 0.0100 ± 0.0036 | 0.11 ± 0.04 |
| MAN | 0.012 | 0.0011 | 69.12 ± 2.51 | 27.31 ± 1.74 | 23.49 ± 3.64 | 57.47 ± 14.94 | 0.0077 ± 0.0021 | 0.18 ± 0.03 |
| MAT | 0.015 | 0.0012 | 77.19 ± 4.15 | 81.24 ± 0.71 | 25.62 ± 2.38 | 35.51 ± 1.62 | 0.0073 ± 0.0015 | 0.08 ± 0.05 |
| OMO | 0.012 | 0.0010 | 70.55 ± 1.00 | 78.08 ± 1.62 | 20.11 ± 0.63 | 32.83 ± 3.89 | 0.0060 ± 0.0000 | 0.16 ± 0.01 |
| OPU | 0.012 | 0.0010 | 69.92 ± 2.35 | 81.36 ± 2.67 | 20.89 ± 2.96 | 33.07 ± 2.00 | 0.0067 ± 0.0012 | 0.17 ± 0.03 |
| PAU1 | 0.013 | 0.0012 | 71.57 ± 0.75 | 83.83 ± 1.45 | 27.35 ± 0.89 | 37.79 ± 0.75 | 0.0100 ± 0.0010 | 0.15 ± 0.01 |
| PAU2 | 0.014 | 0.0013 | 72.75 ± 1.48 | 82.71 ± 1.75 | 21.76 ± 1.03 | 38.86 ± 2.66 | 0.0073 ± 0.0012 | 0.14 ± 0.02 |
| POU | 0.012 | 0.0012 | 69.50 ± 2.02 | 80.83 ± 3.33 | 20.58 ± 0.85 | 37.09 ± 3.63 | 0.0067 ± 0.0006 | 0.17 ± 0.02 |
| PUR | 0.013 | 0.0012 | 70.89 ± 1.88 | 81.55 ± 1.36 | 25.18 ± 4.44 | 34.31 ± 1.32 | 0.0080 ± 0.0020 | 0.16 ± 0.02 |
| RAK | 0.016 | 0.0017 | 78.93 ± 1.20 | 85.95 ± 1.42 | 30.75 ± 0.21 | 45.59 ± 2.71 | 0.0097 ± 0.0006 | 0.06 ± 0.01 |
| RAN | 0.014 | 0.0012 | 73.84 ± 1.58 | 83.49 ± 1.83 | 26.02 ± 1.70 | 38.18 ± 4.41 | 0.0083 ± 0.0006 | 0.12 ± 0.02 |
| REA | 0.014 | 0.0011 | 75.68 ± 2.48 | 81.41 ± 1.93 | 24.22 ± 1.06 | 36.00 ± 1.14 | 0.0073 ± 0.0006 | 0.10 ± 0.03 |
| TAH | 0.015 | 0.0010 | 76.25 ± 1.53 | 80.90 ± 0.76 | 25.72 ± 1.44 | 33.37 ± 2.26 | 0.0077 ± 0.0006 | 0.09 ± 0.02 |
| TAI1 | 0.012 | 0.0010 | 69.71 ± 1.41 | 83.31 ± 0.57 | 27.04 ± 2.66 | 30.42 ± 3.33 | 0.0093 ± 0.0012 | 0.18 ± 0.01 |
| TAI2 | 0.013 | 0.0011 | 70.34 ± 2.68 | 80.38 ± 1.63 | 23.18 ± 2.25 | 34.75 ± 0.59 | 0.0090 ± 0.0017 | 0.15 ± 0.01 |
| TBB | 0.009 | 0.0011 | 65.94 ± 1.36 | 71.54 ± 2.50 | 30.37 ± 3.08 | 35.42 ± 0.79 | 0.0143 ± 0.0045 | 0.22 ± 0.02 |
| TEK | 0.017 | 0.0017 | 80.04 ± 1.21 | 82.73 ± 2.73 | 31.57 ± 1.39 | 45.18 ± 1.50 | 0.0103 ± 0.0006 | 0.05 ± 0.01 |
| TRB | 0.012 | 0.0010 | 69.99 ± 1.19 | 75.35 ± 3.17 | 24.49 ± 1.98 | 31.09 ± 8.57 | 0.0077 ± 0.0006 | 0.17 ± 0.01 |
| TUA | 0.014 | 0.0012 | 73.79 ± 2.22 | 81.56 ± 0.17 | 25.52 ± 2.32 | 35.86 ± 1.15 | 0.0083 ± 0.0012 | 0.12 ± 0.03 |
| TYE | 0.015 | 0.0009 | 75.59 ± 1.88 | 80.60 ± 1.59 | 25.69 ± 1.53 | 33.24 ± 1.22 | 0.0080 ± 0.0010 | 0.10 ± 0.02 |
| WEB | 0.015 | 0.0011 | 80.75 ± 1.85 | 80.81 ± 1.76 | 31.67 ± 1.87 | 34.13 ± 3.55 | 0.0097 ± 0.0015 | 0.04 ± 0.00 |
| WHA1 | 0.018 | 0.0012 | 74.57 ± 0.85 | 85.66 ± 0.40 | 26.07 ± 1.32 | 45.54 ± 1.66 | 0.0083 ± 0.0012 | 0.11 ± 0.01 |
| WHA2 | 0.014 | 0.0017 | 68.78 ± 2.01 | 81.98 ± 1.21 | 20.59 ± 1.55 | 32.5 0 ± 3.39 | 0.0080 ± 0.0020 | 0.18 ± 0.02 |
| WHA3 | 0.012 | 0.0010 | 71.86 ± 2.92 | 84.67 ± 3.85 | 25.15 ± 1.77 | 41.16 ± 2.54 | 0.0083 ± 0.0012 | 0.15 ± 0.03 |
| WHI | 0.013 | 0.0015 | 74.56 ± 1.96 | 82.47 ± 1.45 | 24.65 ± 3.57 | 37.29 ± 0.79 | 0.0080 ± 0.0026 | 0.10 ± 0.03 |


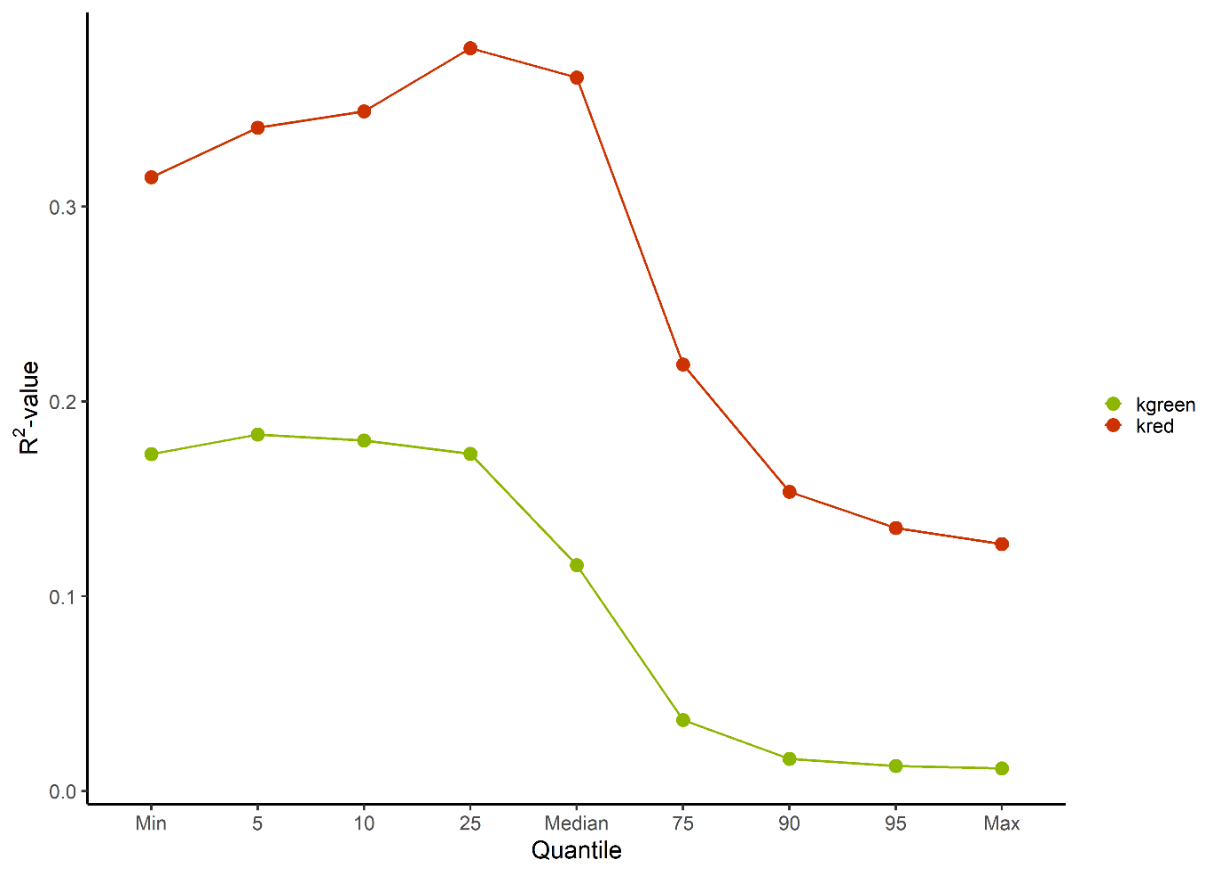


Fig. S1: The coefficient of determination (R^2^) of different temperature quantiles on the degradation rate green (k_green_) and red (k_red_) tea. Individual linear models were fitted to each quantile to establish the coefficient of determination of the relationship.


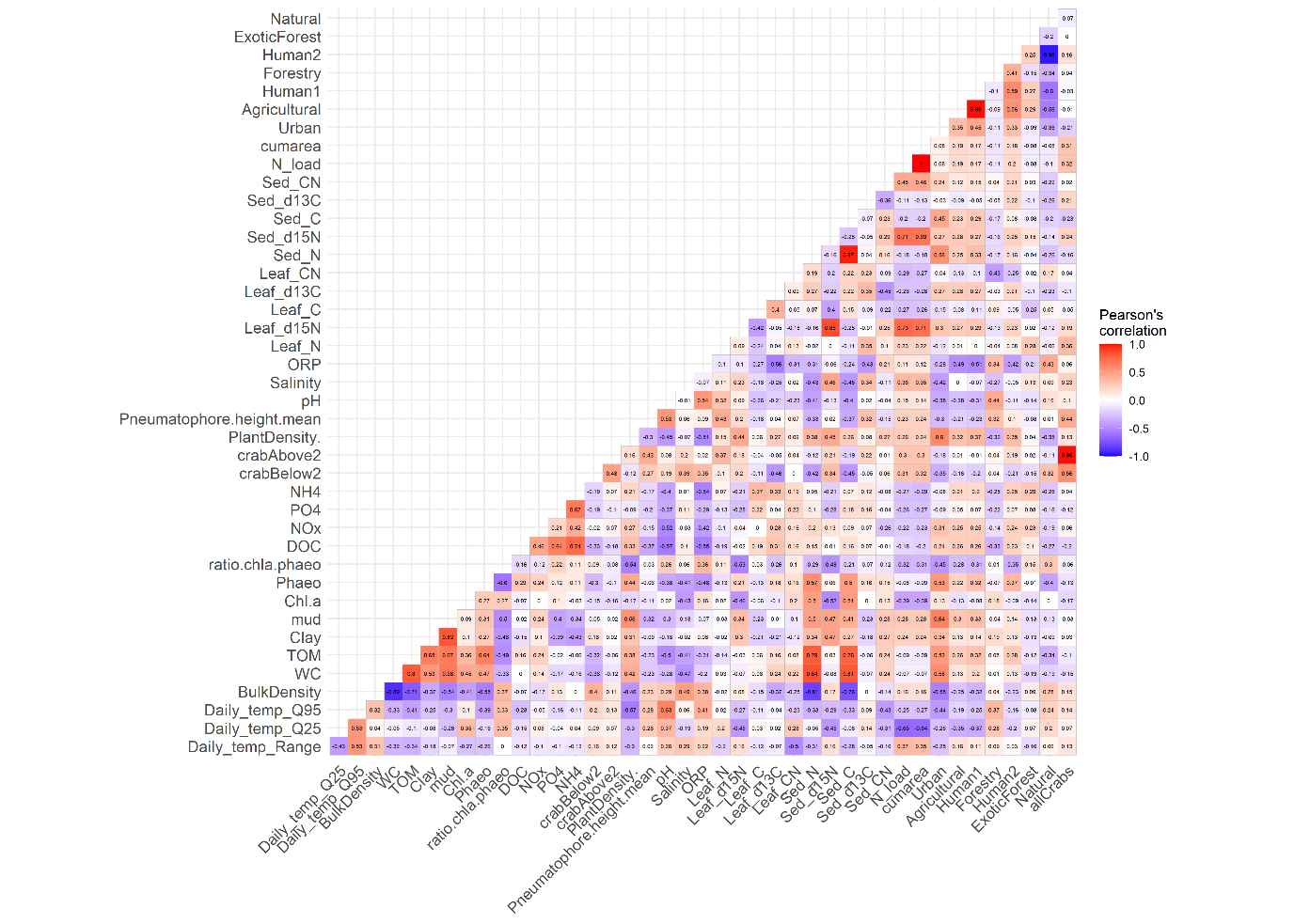


Fig. S2: Correlation matrix of all environmental variables.

Table S2: Measured environmental variable. Those shaded in grey were removed prior to the analysis to avoid collinearity and the correlated variables are shown in the last column. The remaining 23 variables were carried forward into the statistical analysis. Notations used in the text as well as the units are given.

| **Variable** | **Abbreviation** | **Unit** | **Correlation (R > 0.75)** |
| --- | --- | --- | --- |
| Daily temperature (25^th^) | T_Q25_ | °C |  |
| Human land use in catchment | %Human land use | % |  |
| Tree density |  | % |  |
| Pneumatophore height |  | cm |  |
| Crab burrow density (<2cm) | Crab burrows <2cm | m^2^ |  |
| Crab burrow density (>2cm) | Crab burrows >2cm | m^2^ |  |
| Foliar carbon | Foliar C | % |  |
| Foliar δ^13^C |  | ‰ |  |
| Foliar nitrogen | Foliar N | % |  |
| Foliar δ^15^N |  | ‰ |  |
| Foliar carbon to nitrogen ratio | Foliar C:N |  |  |
| pH |  |  |  |
| Salinity |  |  |  |
| Oxygen reduction potential | ORP | mV |  |
| Sediment bulk density |  | g cm^-3^ | Sed C |
| Grain size | GS | µm | Mud content |
| Sediment water content | WC | % | Sed C |
| Mud content |  | % |  |
| Clay content |  | % | Mud content |
| Total organic matter | TOM | % | Sed C |
| Chlorophyll *a* | Chl a | µg g^-1^ DW |  |
| Phaeopigments | Phaeo | µg g^-1^ DW | Chl a:Phaeo |
| Chlorophyll *a* to Phaeopigment ratio | Chl a: Phaeo |  |  |
| Sediment carbon | Sed C | % |  |
| Sediment δ^13^C | Sed δ^13^C | ‰ |  |
| Sediment nitrogen | Sed N | % | Sed C |
| Sediment δ^15^N | Sed δ^15^N | ‰ | Foliar δ^15^N |
| Sediment carbon to nitrogen ratio | Sed C:N |  |  |
| Dissolved organic carbon | DOC | ppm |  |
| Porewater nitrate and nitrite | NO_x_ | µmol N L^-1^ |  |
| Porewater ammonium | NH_4_^+^ | µmol N L^-1^ |  |
| Porewater ortho-phosphate | PO_4_^3-^ | µmol P L^-1^ | DOC |

Table S3: Marginal effects of the ten most important individual predictors of the degradation of green (k_green_) and red (k_red_) tea.

| **k_green_** | **R^2^** | **k_red_** | **R^2^** |
| --- | --- | --- | --- |
| NO_x_ | 0.22 | T_Q25_ | 0.38 |
| T_Q25_ | 0.17 | Crab burrows (>2 cm) | 0.16 |
| ORP | 0.15 | Pneumatophore height | 0.14 |
| SOC:N | 0.13 | Crab burrows (<2 cm) | 0.13 |
| Foliar δ^13^C | 0.10 | Foliar C:N | 0.09 |
| Foliar C:N | 0.08 | pH | 0.08 |
| Chl *a* | 0.06 | Sediment δ^13^C | 0.06 |
| Sediment δ^13^C | 0.06 | SOC:N | 0.05 |
| %Human land use | 0.06 | Foliar C | 0.04 |
| Tree density | 0.05 | Chl *a* | 0.04 |

Table S4: Relative importance of predictor variables controlling green tea decomposition (k_green_) in temperate mangrove forests as calculated from the random forest model. Variables are ranked by importance within the model. Mean and standard deviation were calculated using 100 bootstraps.

| **Rank** | **Variable** | **variable importance  Mean** | **variable importance  SD** |
| --- | --- | --- | --- |
| 1 | Foliar δ^15^N | 13.39 | 4.35 |
| 2 | Foliar C:N | 7.87 | 1.87 |
| 3 | Salinity | 7.52 | 1.34 |
| 4 | %Human land use | 6.40 | 1.20 |
| 5 | Sediment C:N | 6.24 | 1.23 |
| 6 | NO_x_ | 6.23 | 1.68 |
| 7 | Sediment C | 6.11 | 1.23 |
| 8 | Sediment δ^13^C | 5.80 | 0.94 |
| 9 | ORP | 5.76 | 0.91 |
| 10 | Foliar C | 5.59 | 0.93 |
| 11 | Pneumatophore height | 5.44 | 0.95 |
| 12 | Chlorophyll a | 5.44 | 1.31 |
| 13 | Foliar δ^13^C | 5.40 | 1.22 |
| 14 | Foliar N | 5.31 | 0.90 |
| 15 | T_Q25_ | 5.21 | 0.92 |
| 16 | pH | 4.78 | 0.84 |
| 17 | Chorophyll a:Phaeophytin | 4.30 | 0.80 |
| 18 | Tree density | 4.26 | 0.98 |
| 19 | Mud content | 4.16 | 0.68 |
| 20 | Crab burrows (<2 cm) | 3.58 | 0.92 |
| 21 | DOC | 3.17 | 0.70 |
| 22 | NH_4_ | 3.05 | 0.83 |
| 23 | Crab burrows (>2 cm) | 2.68 | 0.57 |

Table S5: Relative importance of predictor variables controlling red tea decomposition (k_red_) in temperate mangrove forests as calculated from the random forest model. Variables are ranked by importance within the model. Mean and standard deviation were calculated using 100 bootstraps.

| **Rank** | **Variable** | **variable importance  Mean** | **variable importance  SD** |
| --- | --- | --- | --- |
| 1 | T_Q25_ | 14.66 | 4.88 |
| 2 | pH | 10.21 | 3.09 |
| 3 | Sediment C | 8.61 | 2.05 |
| 4 | Foliar C:N | 8.48 | 2.28 |
| 5 | Foliar N | 8.16 | 3.29 |
| 6 | %Human land use | 7.14 | 2.28 |
| 7 | Foliar δ^15^N | 7.07 | 1.64 |
| 8 | Sediment C:N | 5.96 | 2.12 |
| 9 | Foliar C | 5.71 | 1.40 |
| 10 | Sediment δ^13^C | 5.44 | 1.75 |
| 11 | Foliar δ^13^C | 5.01 | 1.24 |
| 12 | Pneumatophore height | 4.69 | 1.28 |
| 13 | ORP | 4.59 | 1.31 |
| 14 | Tree density | 4.34 | 1.47 |
| 15 | Mud content | 4.03 | 0.93 |
| 16 | Crab burrows (<2 cm) | 3.90 | 1.61 |
| 17 | Crab burrows (>2 cm) | 3.79 | 1.52 |
| 18 | Salinity | 3.60 | 1.20 |
| 19 | NO_x_ | 3.27 | 1.27 |
| 20 | NH_4_ | 2.93 | 0.91 |
| 21 | Chlorophyll a | 2.67 | 0.62 |
| 22 | DOC | 2.63 | 0.97 |
| 23 | Chlorophyll:Phaeophytin | 2.60 | 0.84 |


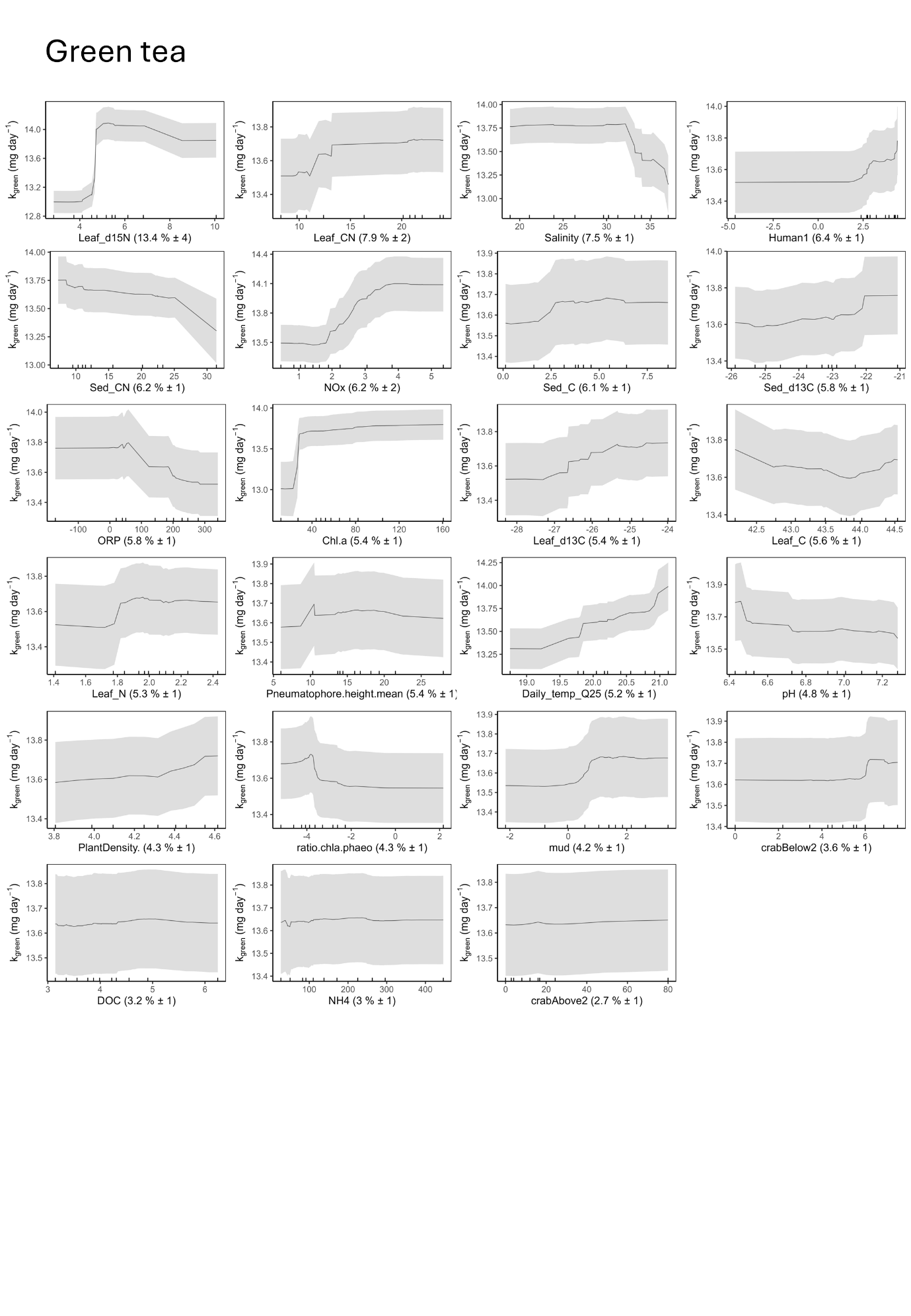


Fig. S3: Partial dependence plots of all variables’ relationship with degradation rate of green tea (k_green_) ordered by relative importance to the model performance. Black lines are means and ribbons represent the standard deviation from 100 bootstrap iterations.


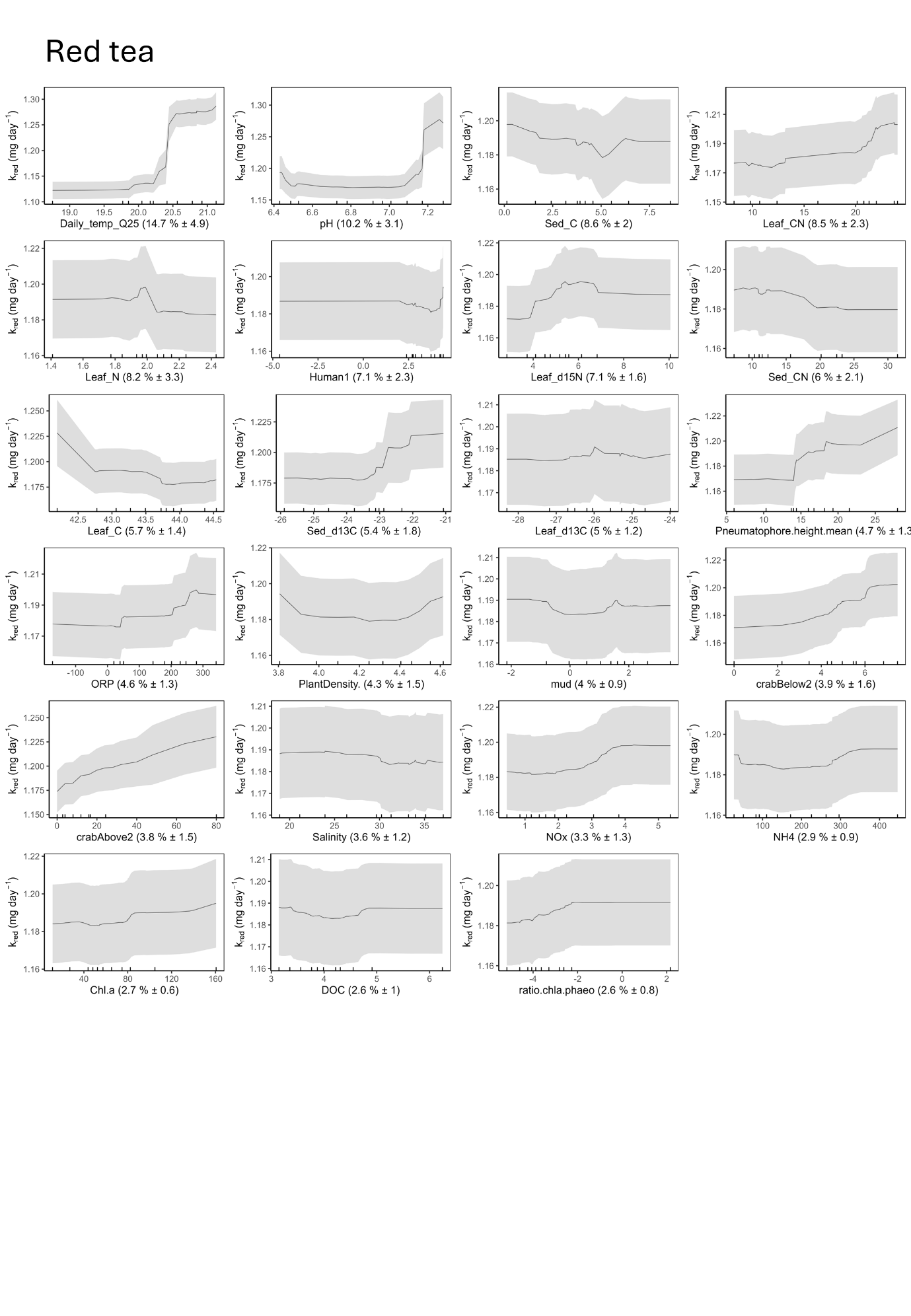


Fig. S4: Partial dependence plots of all variables’ relationship with degradation rate of red tea (k_red_) ordered by relative importance to the model performance. Black lines are means and ribbons represent the standard deviation from 100 bootstrap iterations.


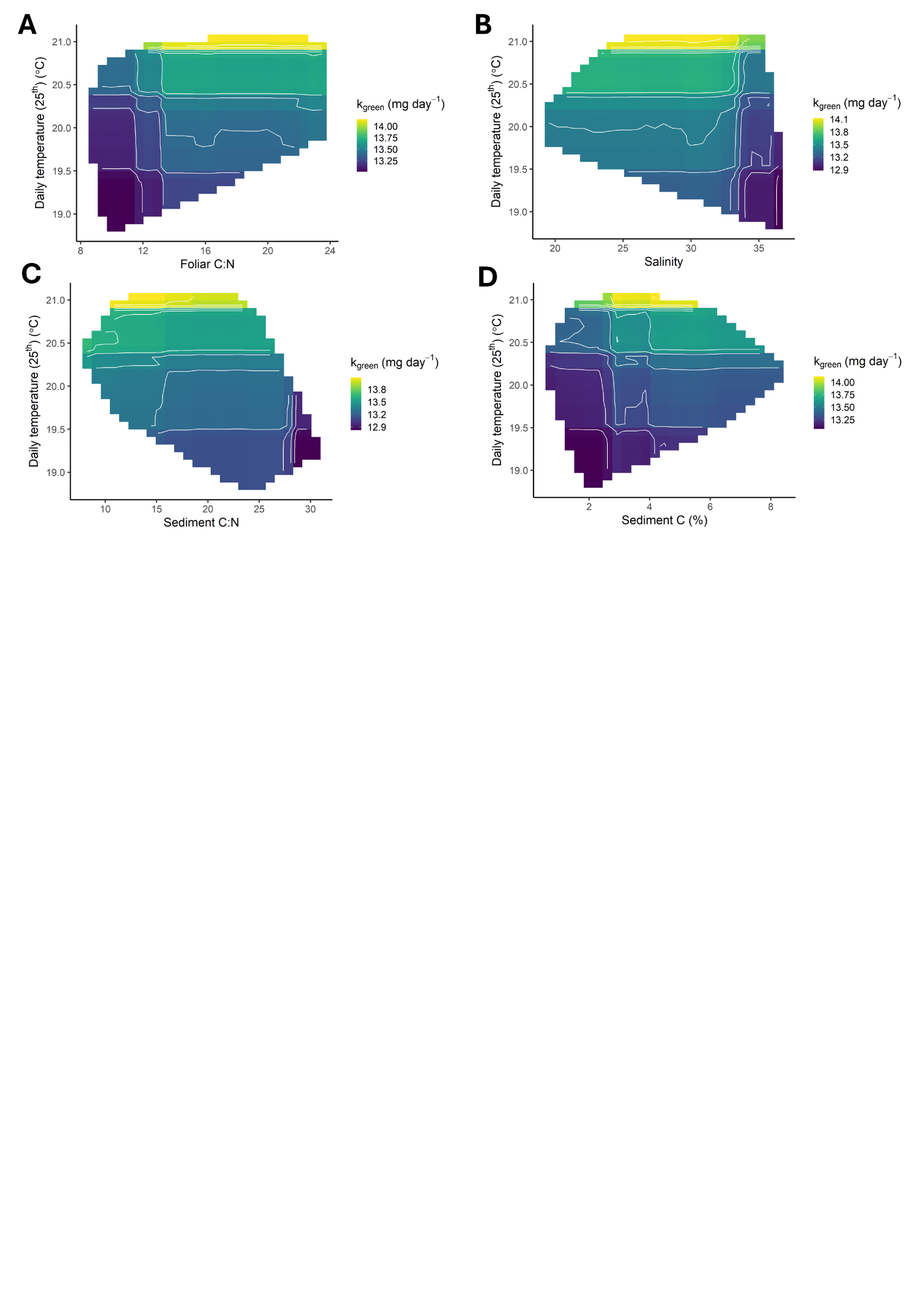


Fig. S5: Human land use induced effects on degradation of labile organic matter (green tea) in temperate mangrove forests. Two-variable partial dependence plots of daily temperature (25^th^ quantile) with **A** foliar C:N, **B** salinity, **C** sediment C:N, and **D** sediment C.


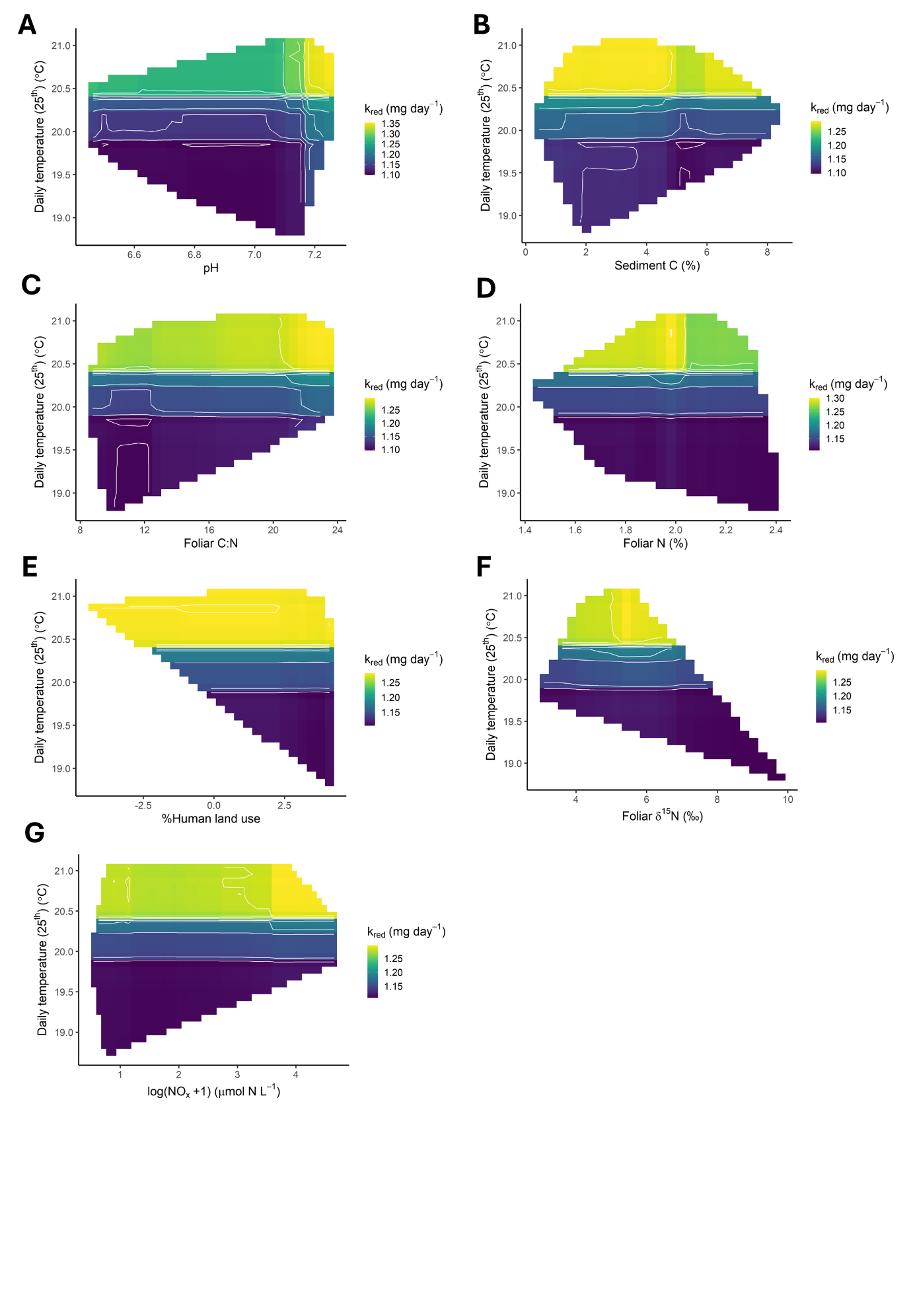


Fig. S6: Human land use induced effects on degradation of recalcitrant organic matter (red tea) in temperate mangrove forests. Two-variable partial dependence plots of daily temperature (25^th^ quantile) with **A** pH, **B** sediment C, **C** foliar C:N, **D** foliar N, **E** %Human land use, **F** foliar δ^15^, and **G** nitrate/nitrite concentrations.
